# Supplementary material for: Investigating the effects of chimerism on the inference of selection: quantifying genomic targets of purifying, positive, and balancing selection in common marmosets (Callithrix jacchus)
Source: Heredity (Edinb). 2025 Oct 8;134(10-11):645–57. doi: 10.1038/s41437-025-00804-7 (PMC12577292; doi:10.1038/s41437-025-00804-7)
Supplement: Supplementary file 1 — Supplementary Tables and Figures [file 41437_2025_804_MOESM1_ESM.pdf]

Supplementary Table S1

Analysis	Category	Term	*p*-value	Fold Enrichment	FDR
All selective sweep candidates	UP_SEQ_FEATURE	COMPBIAS:Basic and acidic residues	3.09E-06	1.483	0.003
	UP_SEQ_FEATURE	REGION:Disordered	6.54E-05	1.185	0.035
	UP_SEQ_FEATURE	COMPBIAS:Polar residues	1.33E-04	1.429	0.038
	UP_SEQ_FEATURE	COMPBIAS:Basic residues	1.44E-04	1.778	0.038
	UP_KW_PTM	KW-0597~Phosphoprotein	2.96E-04	1.234	0.006
	UP_KW_PTM	KW-0007~Acetylation	5.64E-04	1.507	0.006
	UP_KW_CELLULAR_COMPONENT	KW-0333~Golgi apparatus	9.12E-04	2.164	0.035
Peak selective sweep genes	UP_KW_CELLULAR_COMPONENT	KW-0539~Nucleus	1.97E-03	1.870	0.034
Peak selective sweep genes with high mean divergence	UP_SEQ_FEATURE	CARBOHYD:N-linked (GlcNAc...) asparagine	5.53E-07	1.373	0.002
	GOTERM_CC_DIRECT	GO:0062023~collagen-containing extracellular matrix	2.66E-05	2.392	0.007
	GOTERM_CC_DIRECT	GO:0016020~membrane	3.84E-05	1.265	0.007
	GOTERM_CC_DIRECT	GO:0005886~plasma membrane	4.29E-05	1.257	0.007
	UP_KW_LIGAND	KW-0106~Calcium	1.16E-04	1.627	0.004
	GOTERM_CC_DIRECT	GO:0005576~extracellular region	1.33E-04	1.427	0.017
	UP_KW_PTM	KW-0325~Glycoprotein	2.24E-04	1.221	0.006
	UP_KW_CELLULAR_COMPONENT	KW-0966~Cell projection	8.31E-04	1.491	0.027

Supplementary Table S1: Enriched gene categories from functional analysis with DAVID, with false discovery rates (FDR) and *p*-values <0.05 for all selective sweep candidate genes (top), genes at likelihood surface peaks (middle), and genes that have both high divergence and are at likelihood surface peaks (bottom).

Supplementary Table S2

chrom	length	# SNPs	# invariant sites	Ts/Tv
1	216,975,769	257,119	47,237,148	2.20
2	202,808,461	222,710	40,777,876	2.11
3	189,455,076	162,252	28,397,479	2.01
4	173,414,057	228,055	37,833,102	2.04
5	161,716,902	213,640	51,551,915	2.16
6	159,674,559	205,517	33,317,930	2.13
7	156,129,252	237,836	43,121,041	2.18
8	126,104,592	169,719	26,637,950	2.10
9	132,900,640	152,330	29,901,412	2.10
10	136,971,485	201,931	33,828,189	2.10
11	128,529,401	178,097	31,238,359	2.13
12	123,697,827	188,874	33,767,759	2.25
13	117,638,787	142,340	29,270,661	2.11
14	112,969,529	149,207	27,988,452	2.09
15	98,621,277	145,110	24,183,383	2.11
16	98,110,366	112,237	19,037,643	1.99
17	74,700,100	88,568	14,898,892	2.03
18	47,063,576	70,276	12,475,879	2.05
19	50,540,917	111,593	15,956,698	2.11
20	44,412,365	76,810	13,152,779	2.29
21	50,614,742	76,637	9,516,850	2.10
22	49,900,457	97,872	12,746,213	2.25
$\sum$ or $\varnothing$	**2,652,950,137**	**3,488,730**	**616,833,610**	**2.12**

Supplementary Table S2: Summary of whole-genome, population-level data.

Supplementary Figure S1

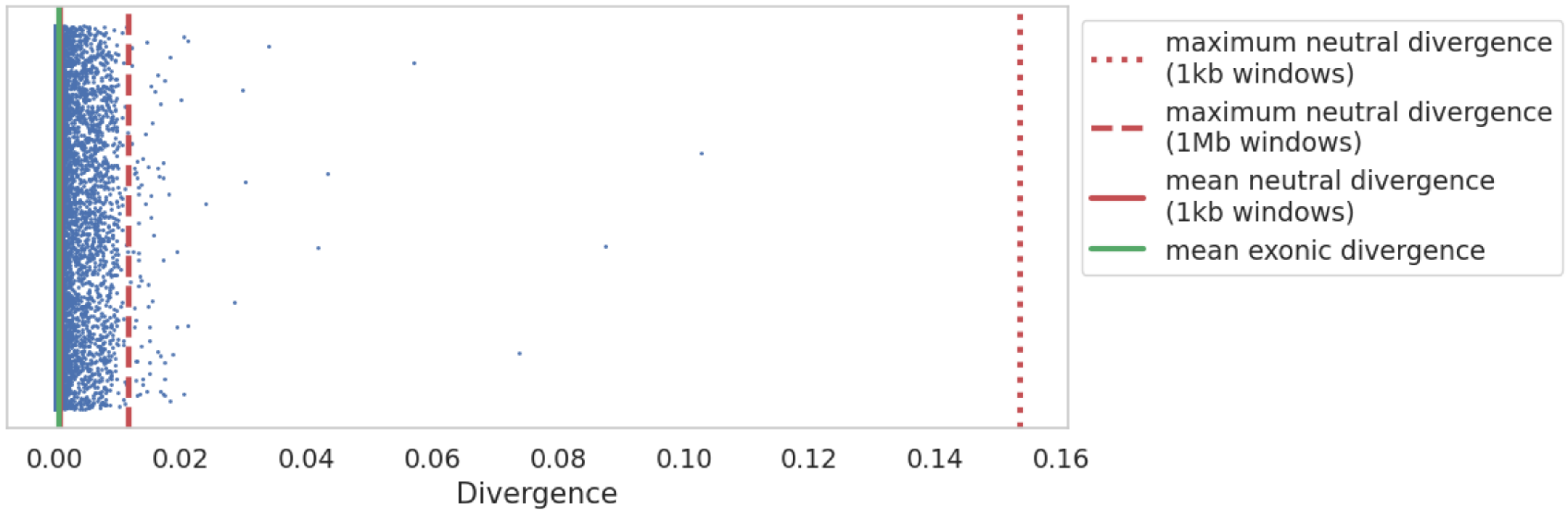

Supplementary Figure S1: Exonic divergence scatter plot with maximum neutral divergence values marked for windows of size 1Mb (red dashed line) and 1kb (red dotted line), as well as the mean neutral divergence for 1kb windows (red solid line), as calculated from Soni et al. (2025b). Each dot represents an autosomal exon, and the mean exonic divergence is plotted (green solid line).

Supplementary Figure S2

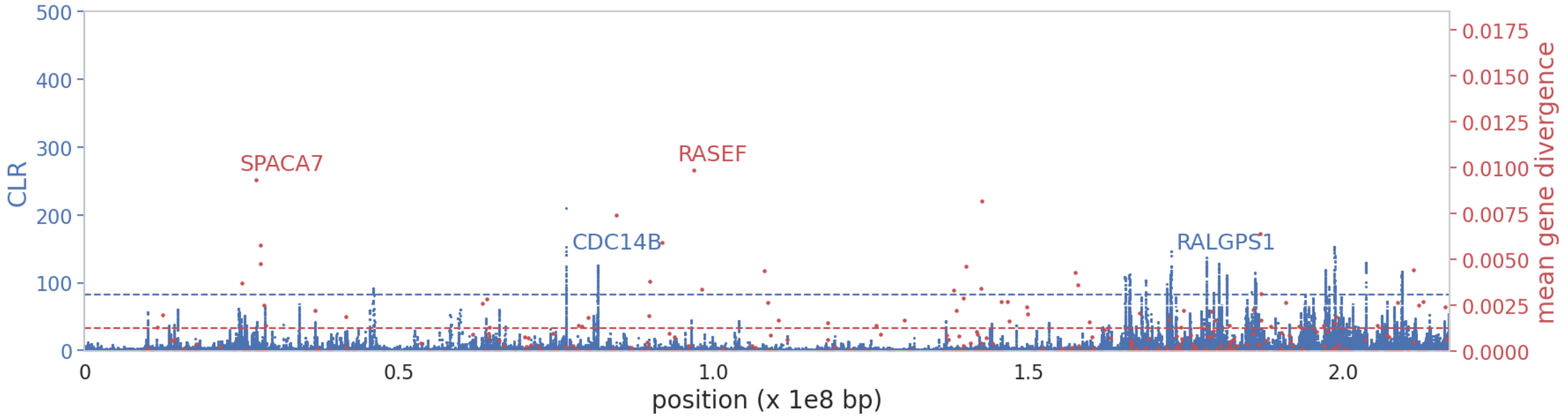

Supplementary Figure S2: SweepFinder2 selective sweep scan results (blue) and exonic divergence (red) for chromosome **1**. The x-axis represents the position along the chromosome, and the y-axis represents the CLR value at each SNP. Blue dashed line represents the null threshold for sweep detection; red dashed line represents 75th percentile neutral divergence. High CLR and highly divergent genes are marked by name.

Supplementary Figure S3

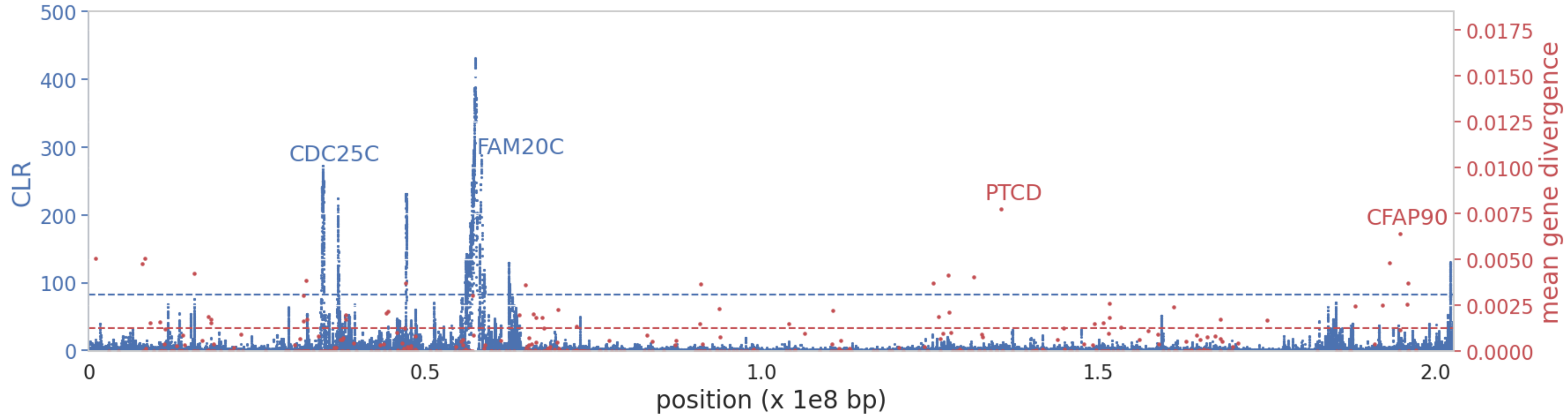

Supplementary Figure S3: SweepFinder2 selective sweep scan results (blue) and exonic divergence (red) for chromosome **2**. The x-axis represents the position along the chromosome, and the y-axis represents the CLR value at each SNP. Blue dashed line represents the null threshold for sweep detection; red dashed line represents 75th percentile neutral divergence. High CLR and highly divergent genes are marked by name.

Supplementary Figure S4

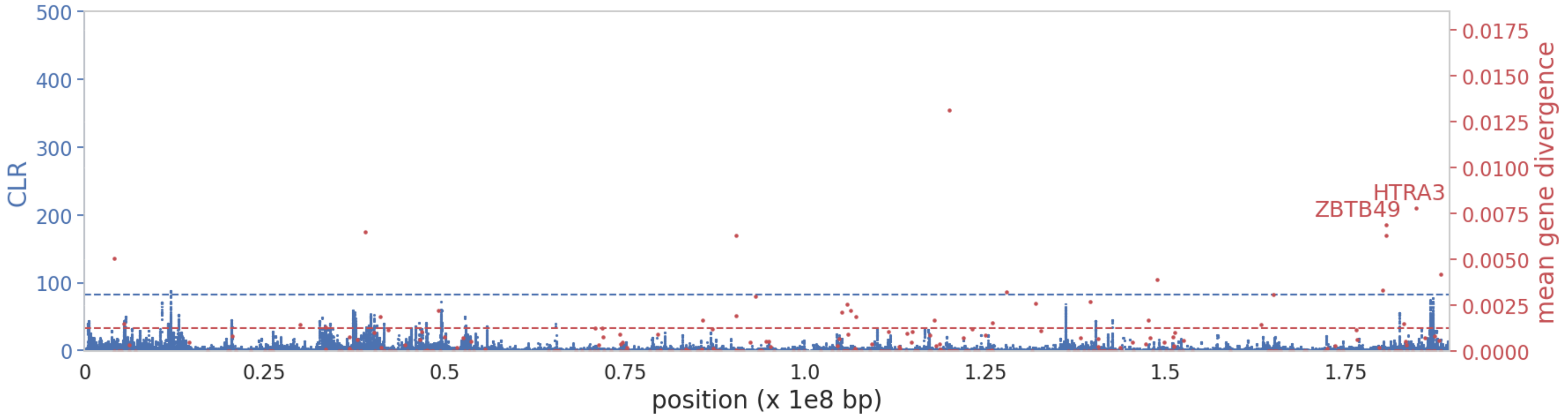

Supplementary Figure S4: SweepFinder2 selective sweep scan results (blue) and exonic divergence (red) for chromosome **3**. The x-axis represents the position along the chromosome, and the y-axis represents the CLR value at each SNP. Blue dashed line represents the null threshold for sweep detection; red dashed line represents 75th percentile neutral divergence. High CLR and highly divergent genes are marked by name.

Supplementary Figure S5

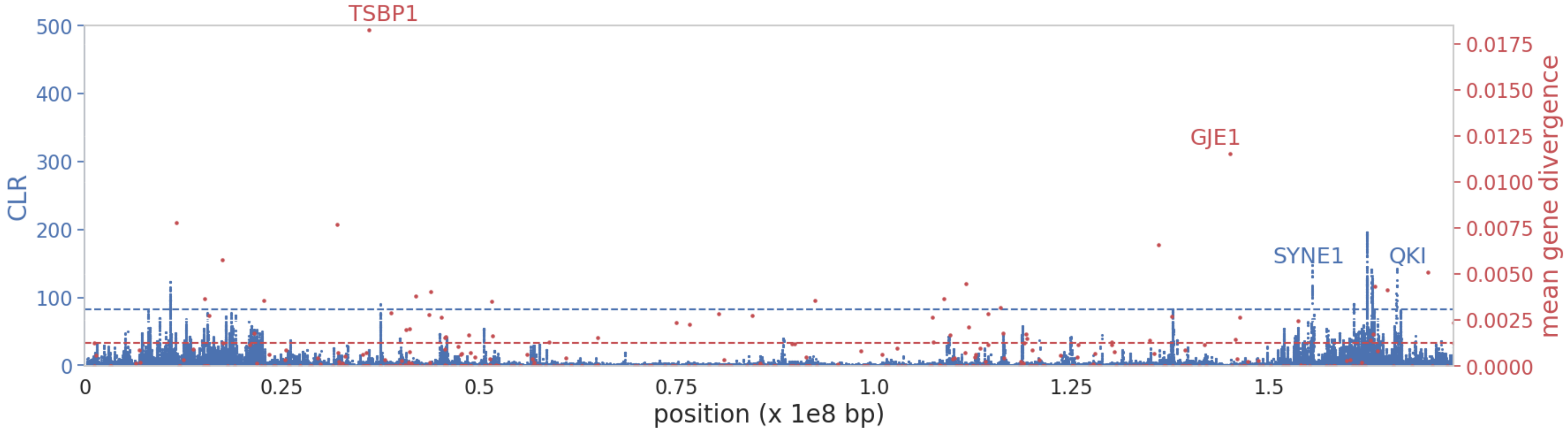

Supplementary Figure S5: SweepFinder2 selective sweep scan results (blue) and exonic divergence (red) for chromosome **4**. The x-axis represents the position along the chromosome, and the y-axis represents the CLR value at each SNP. Blue dashed line represents the null threshold for sweep detection; red dashed line represents 75th percentile neutral divergence. High CLR and highly divergent genes are marked by name.

Supplementary Figure S6

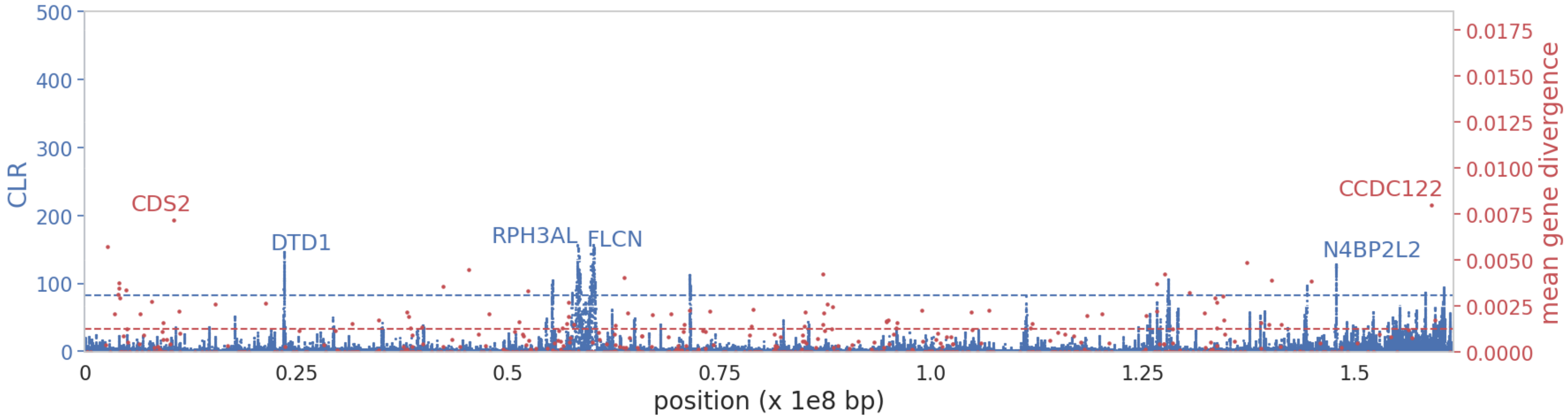

Supplementary Figure S6: SweepFinder2 selective sweep scan results (blue) and exonic divergence (red) for chromosome **5**. The x-axis represents the position along the chromosome, and the y-axis represents the CLR value at each SNP. Blue dashed line represents the null threshold for sweep detection; red dashed line represents 75th percentile neutral divergence. High CLR and highly divergent genes are marked by name.

Supplementary Figure S7

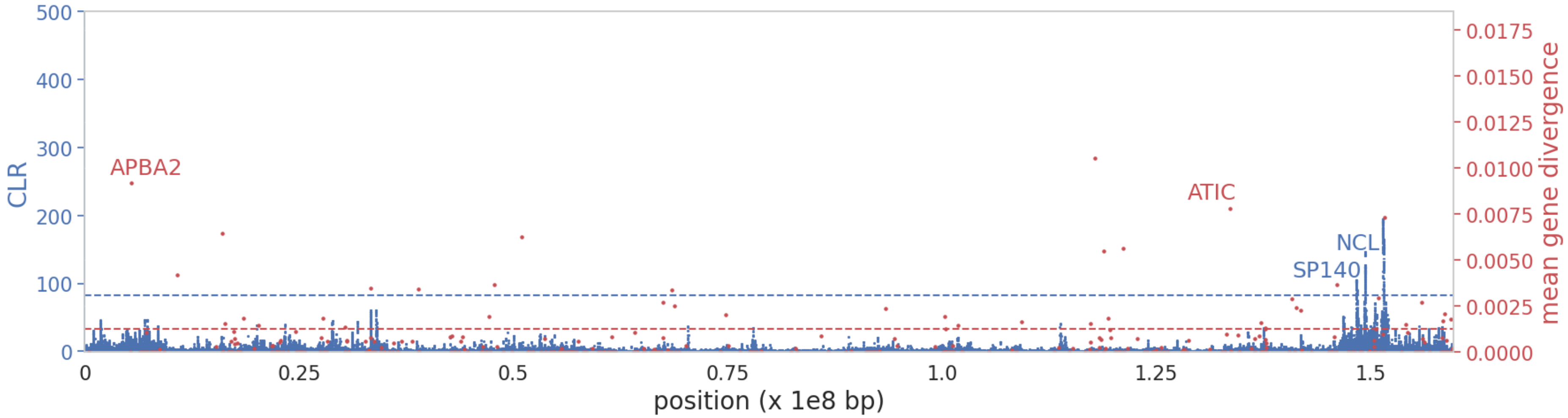

Supplementary Figure S7: SweepFinder2 selective sweep scan results (blue) and exonic divergence (red) for chromosome **6**. The x-axis represents the position along the chromosome, and the y-axis represents the CLR value at each SNP. Blue dashed line represents the null threshold for sweep detection; red dashed line represents 75th percentile neutral divergence. High CLR and highly divergent genes are marked by name.

Supplementary Figure S8

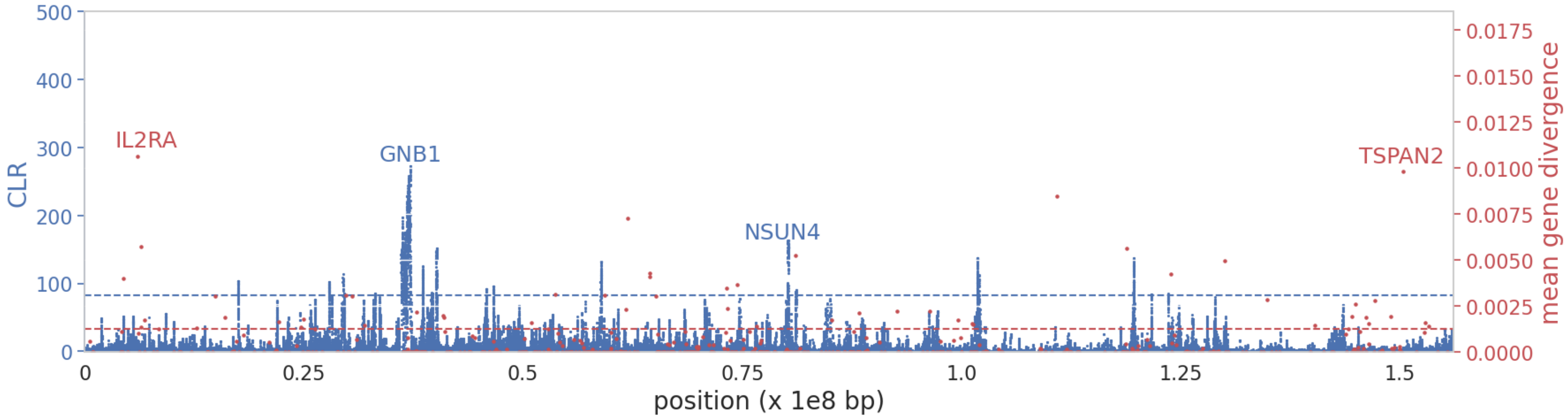

Supplementary Figure S8: SweepFinder2 selective sweep scan results (blue) and exonic divergence (red) for chromosome **7**. The x-axis represents the position along the chromosome, and the y-axis represents the CLR value at each SNP. Blue dashed line represents the null threshold for sweep detection; red dashed line represents 75th percentile neutral divergence. High CLR and highly divergent genes are marked by name.

Supplementary Figure S9

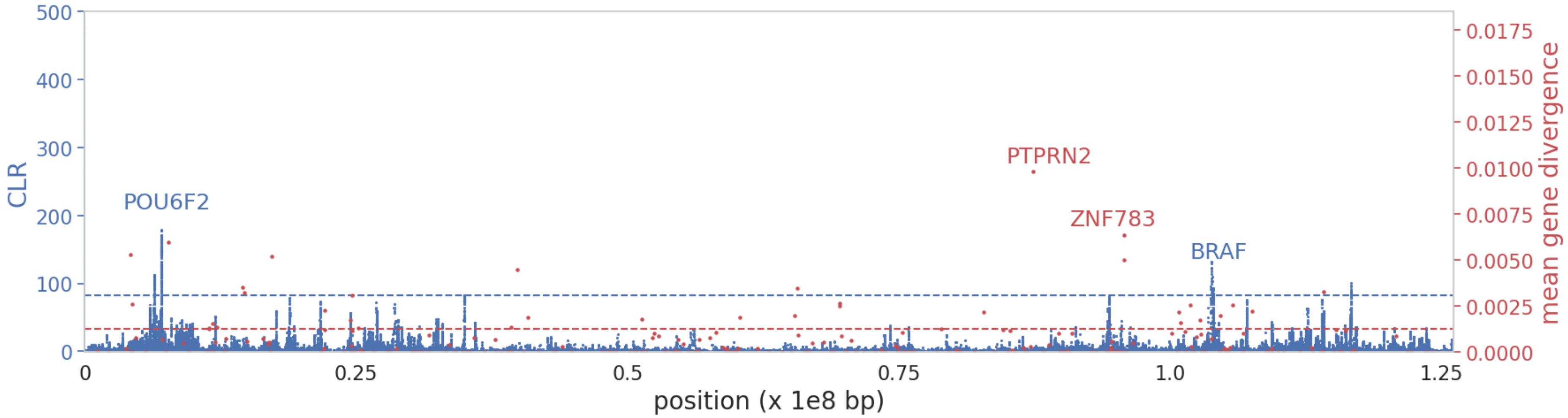

Supplementary Figure S9: SweepFinder2 selective sweep scan results (blue) and exonic divergence (red) for chromosome **8**. The x-axis represents the position along the chromosome, and the y-axis represents the CLR value at each SNP. Blue dashed line represents the null threshold for sweep detection; red dashed line represents 75th percentile neutral divergence. High CLR and highly divergent genes are marked by name.

Supplementary Figure S10

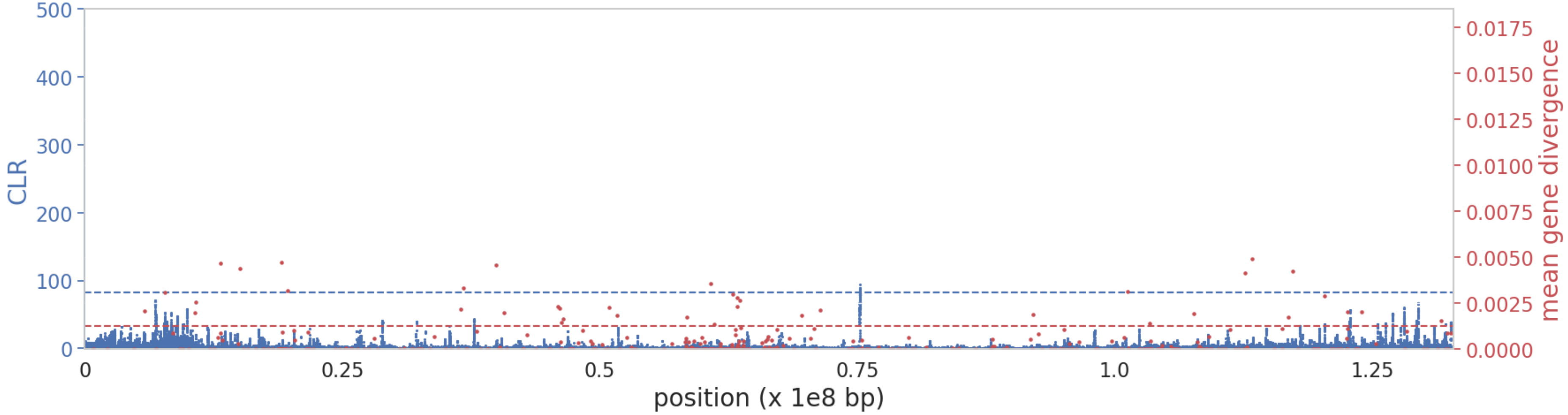

Supplementary Figure S10: SweepFinder2 selective sweep scan results (blue) and exonic divergence (red) for chromosome **9**. The x-axis represents the position along the chromosome, and the y-axis represents the CLR value at each SNP. Blue dashed line represents the null threshold for sweep detection; red dashed line represents 75th percentile neutral divergence. High CLR and highly divergent genes are marked by name.

Supplementary Figure S11

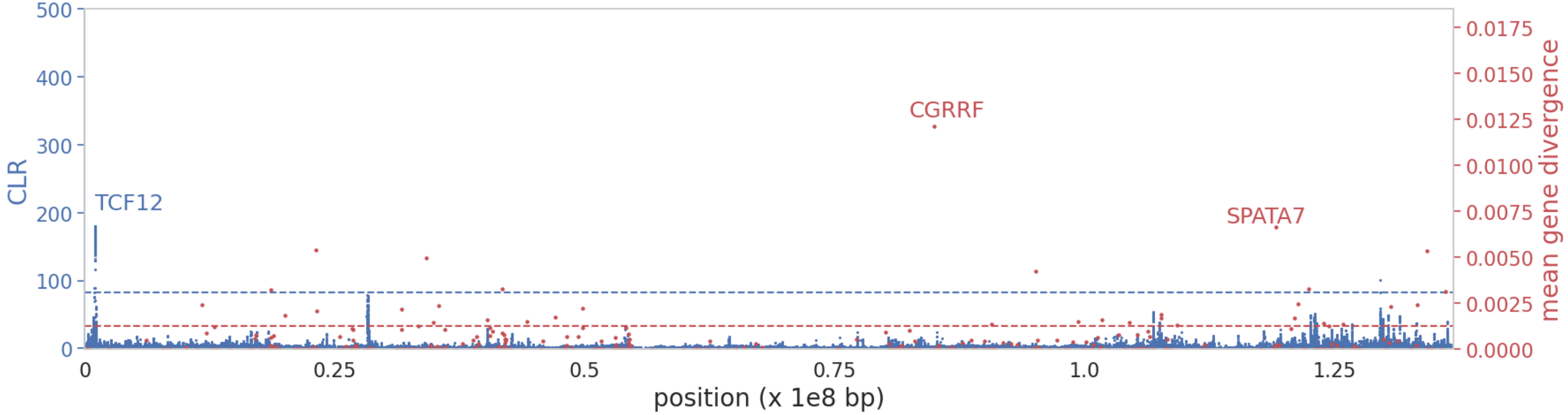

Supplementary Figure S11: SweepFinder2 selective sweep scan results (blue) and exonic divergence (red) for chromosome **10**. The x-axis represents the position along the chromosome, and the y-axis represents the CLR value at each SNP. Blue dashed line represents the null threshold for sweep detection; red dashed line represents 75th percentile neutral divergence. High CLR and highly divergent genes are marked by name.

Supplementary Figure S12

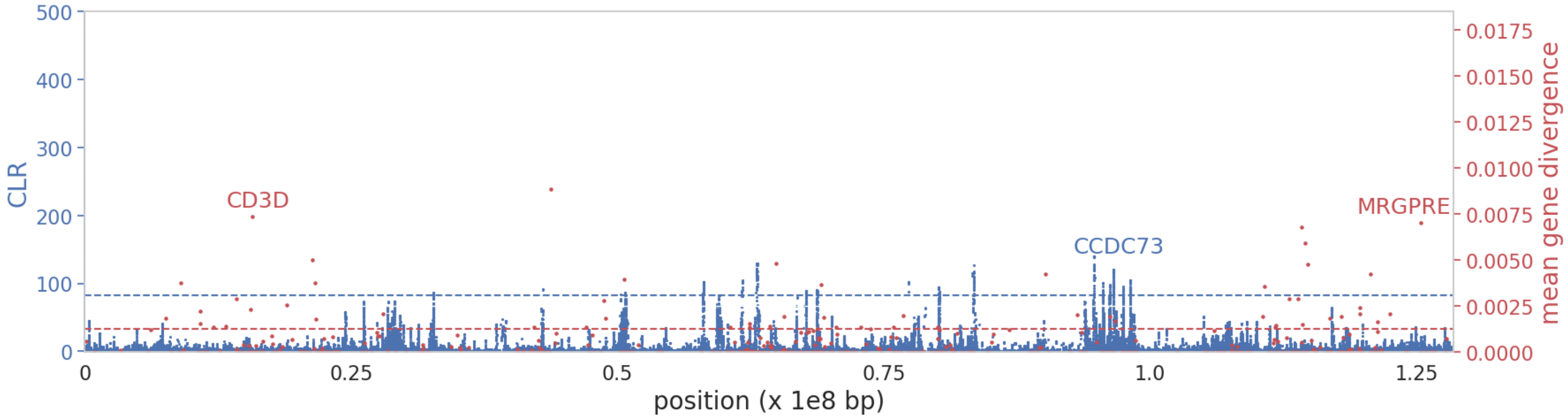

Supplementary Figure S12: SweepFinder2 selective sweep scan results (blue) and exonic divergence (red) for chromosome **11**. The x-axis represents the position along the chromosome, and the y-axis represents the CLR value at each SNP. Blue dashed line represents the null threshold for sweep detection; red dashed line represents 75th percentile neutral divergence. High CLR and highly divergent genes are marked by name.

Supplementary Figure S13

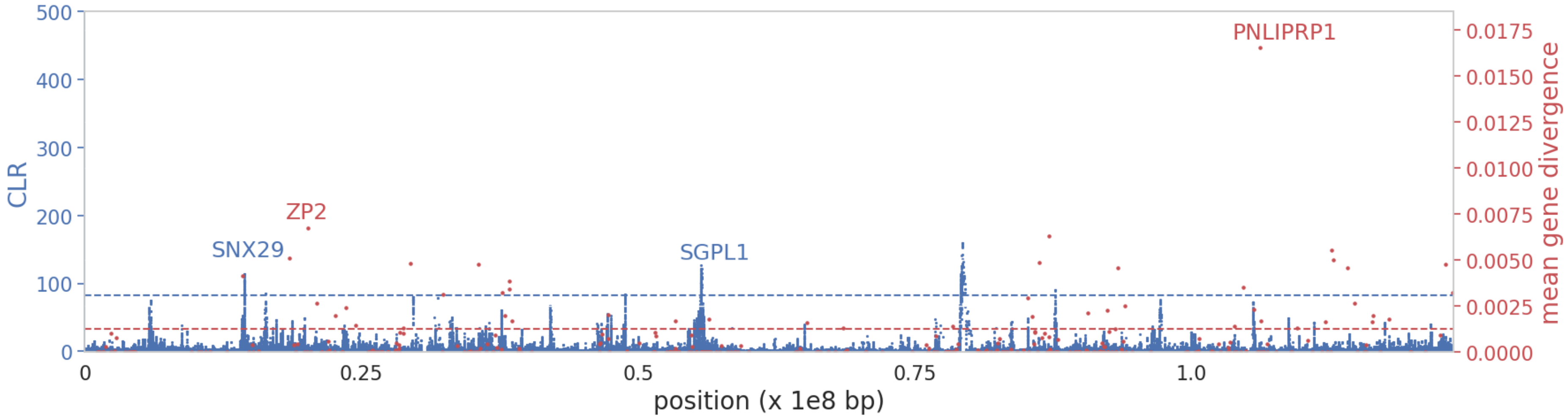

Supplementary Figure S13: SweepFinder2 selective sweep scan results (blue) and exonic divergence (red) for chromosome **12**. The x-axis represents the position along the chromosome, and the y-axis represents the CLR value at each SNP. Blue dashed line represents the null threshold for sweep detection; red dashed line represents 75th percentile neutral divergence. High CLR and highly divergent genes are marked by name.

Supplementary Figure S14

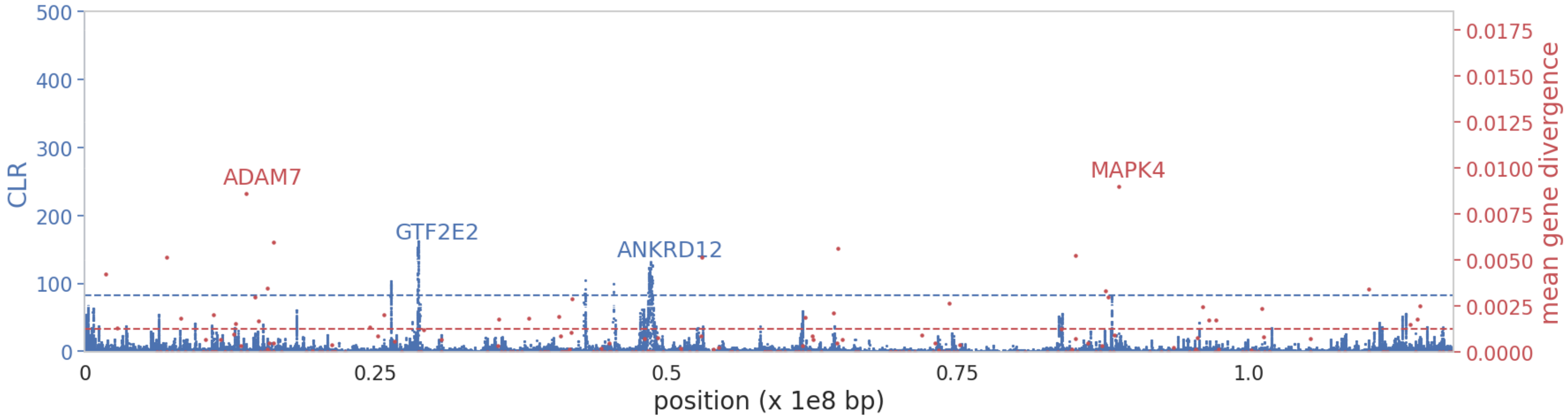

Supplementary Figure S14: SweepFinder2 selective sweep scan results (blue) and exonic divergence (red) for chromosome **13**. The x-axis represents the position along the chromosome, and the y-axis represents the CLR value at each SNP. Blue dashed line represents the null threshold for sweep detection; red dashed line represents 75th percentile neutral divergence. High CLR and highly divergent genes are marked by name.

Supplementary Figure S15

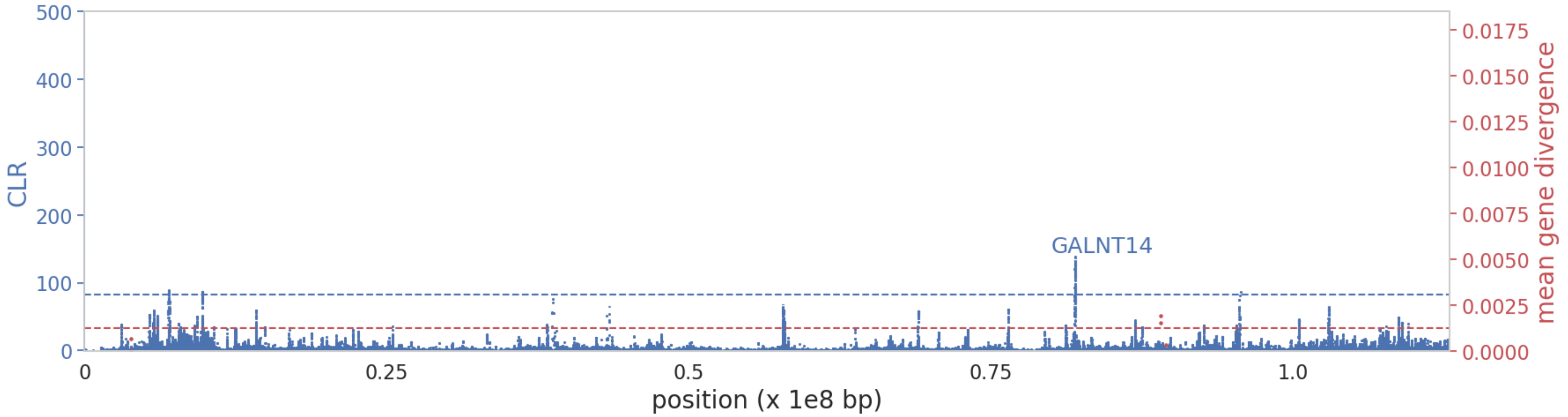

Supplementary Figure S15: SweepFinder2 selective sweep scan results (blue) and exonic divergence (red) for chromosome **14**. The x-axis represents the position along the chromosome, and the y-axis represents the CLR value at each SNP. Blue dashed line represents the null threshold for sweep detection; red dashed line represents 75th percentile neutral divergence. High CLR and highly divergent genes are marked by name.

Supplementary Figure S16

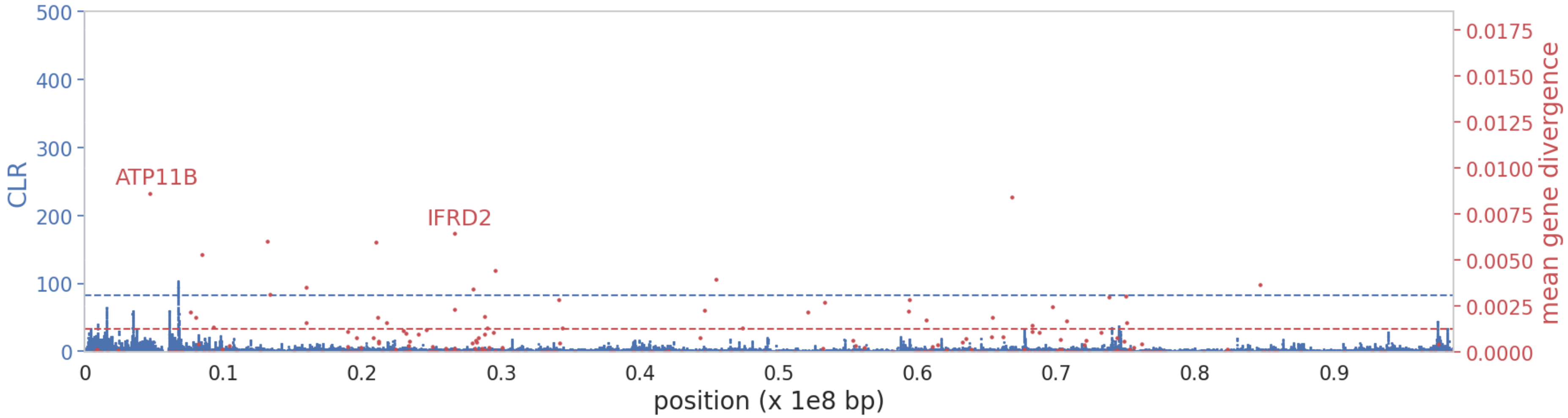

Supplementary Figure S16: SweepFinder2 selective sweep scan results (blue) and exonic divergence (red) for chromosome **15**. The x-axis represents the position along the chromosome, and the y-axis represents the CLR value at each SNP. Blue dashed line represents the null threshold for sweep detection; red dashed line represents 75th percentile neutral divergence. High CLR and highly divergent genes are marked by name.

Supplementary Figure S17

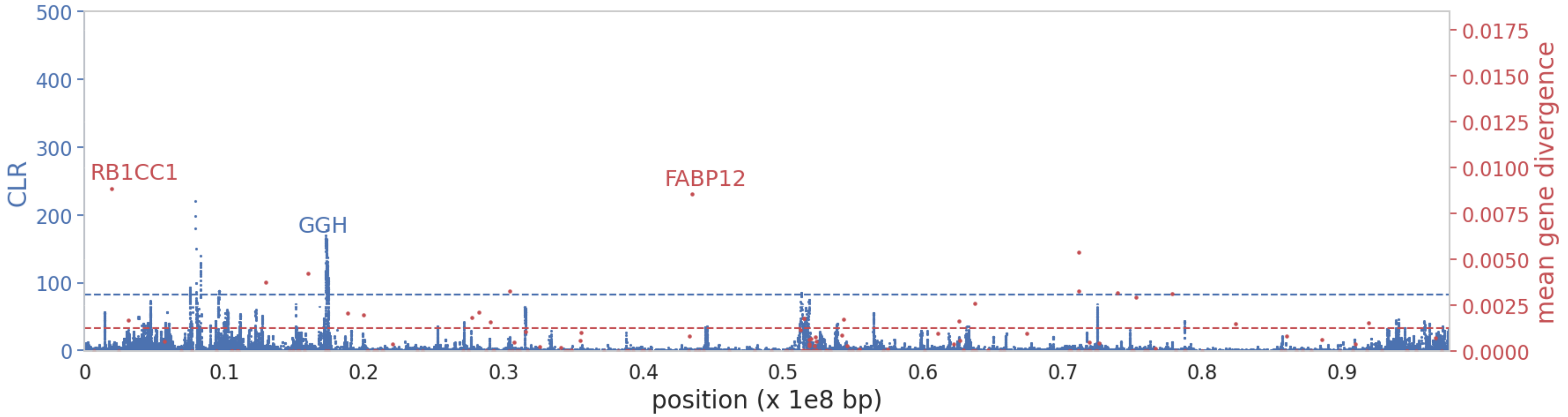

Supplementary Figure S17: SweepFinder2 selective sweep scan results (blue) and exonic divergence (red) for chromosome **16**. The x-axis represents the position along the chromosome, and the y-axis represents the CLR value at each SNP. Blue dashed line represents the null threshold for sweep detection; red dashed line represents 75th percentile neutral divergence. High CLR and highly divergent genes are marked by name.

Supplementary Figure S18

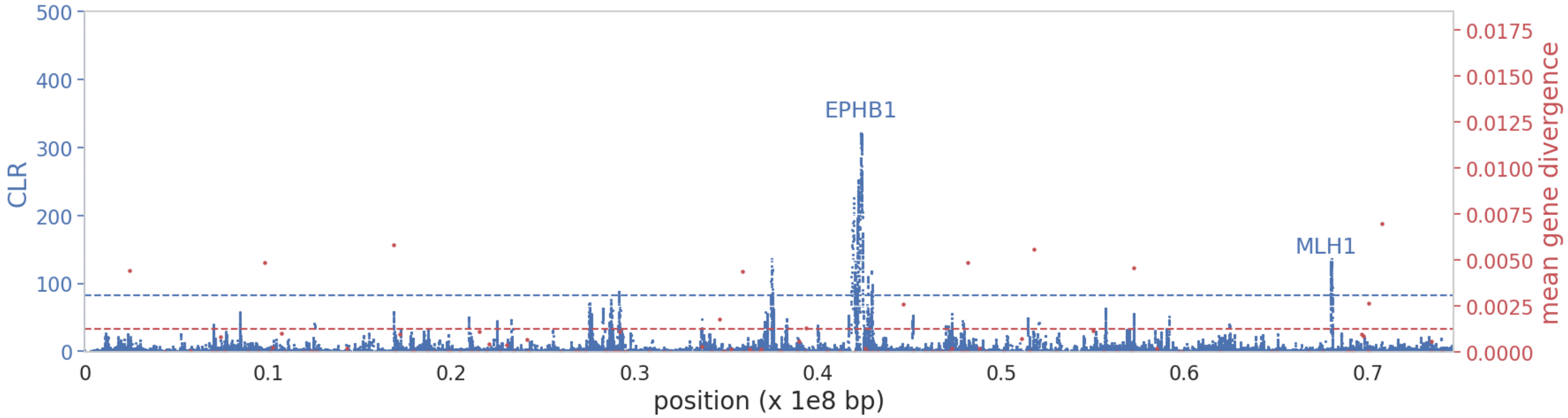

Supplementary Figure S18: SweepFinder2 selective sweep scan results (blue) and exonic divergence (red) for chromosome **17**. The x-axis represents the position along the chromosome, and the y-axis represents the CLR value at each SNP. Blue dashed line represents the null threshold for sweep detection; red dashed line represents 75th percentile neutral divergence. High CLR and highly divergent genes are marked by name.

Supplementary Figure S19

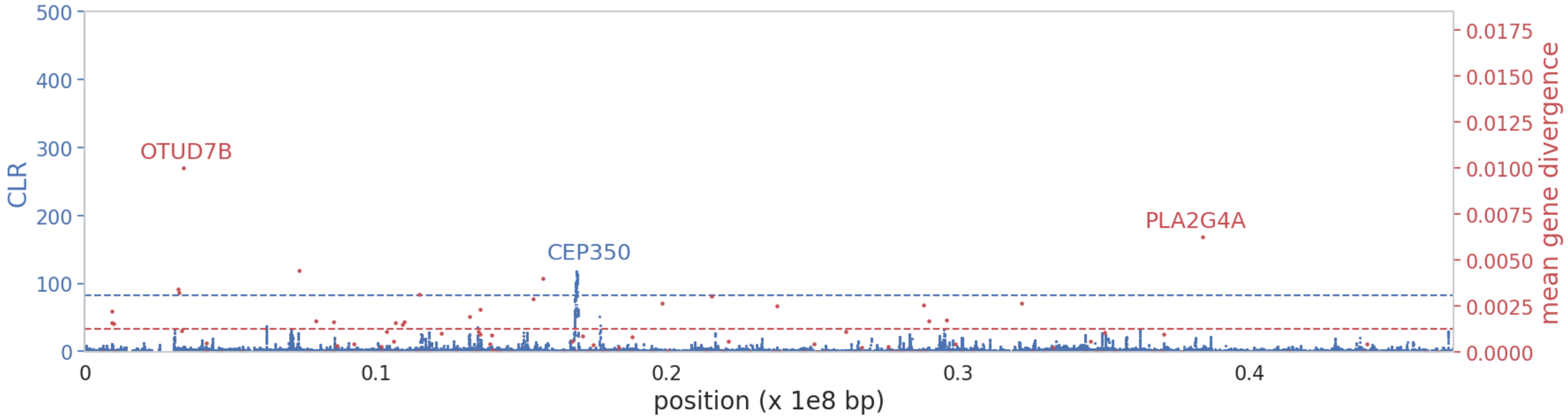

Supplementary Figure S19: SweepFinder2 selective sweep scan results (blue) and exonic divergence (red) for chromosome **18**. The x-axis represents the position along the chromosome, and the y-axis represents the CLR value at each SNP. Blue dashed line represents the null threshold for sweep detection; red dashed line represents 75th percentile neutral divergence. High CLR and highly divergent genes are marked by name.

Supplementary Figure S20

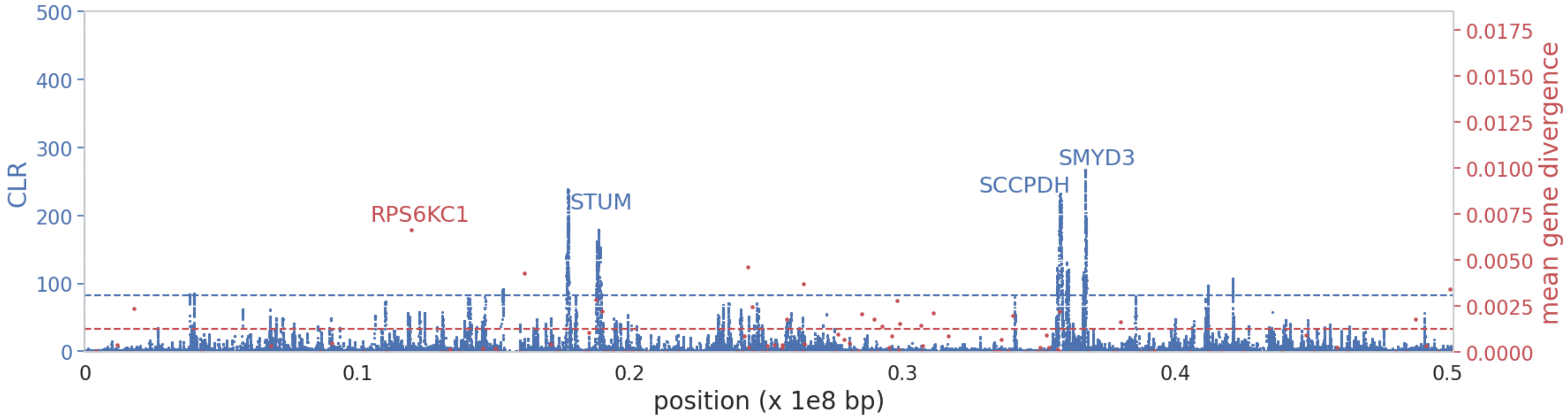

Supplementary Figure S20: SweepFinder2 selective sweep scan results (blue) and exonic divergence (red) for chromosome **19**. The x-axis represents the position along the chromosome, and the y-axis represents the CLR value at each SNP. Blue dashed line represents the null threshold for sweep detection; red dashed line represents 75th percentile neutral divergence. High CLR and highly divergent genes are marked by name.

Supplementary Figure S21

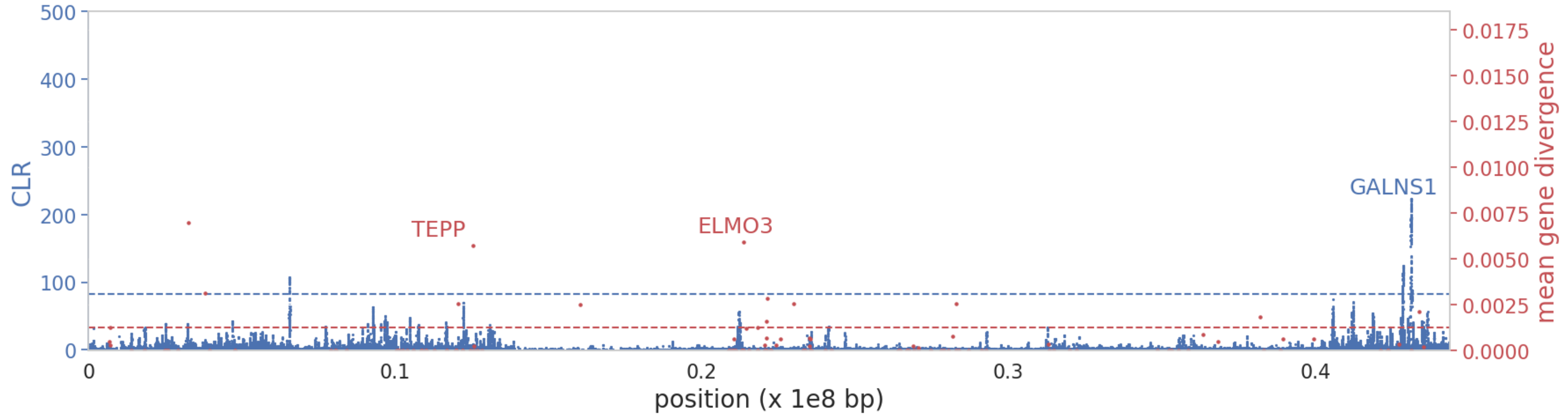

Supplementary Figure S21: SweepFinder2 selective sweep scan results (blue) and exonic divergence (red) for chromosome **20**. The x-axis represents the position along the chromosome, and the y-axis represents the CLR value at each SNP. Blue dashed line represents the null threshold for sweep detection; red dashed line represents 75th percentile neutral divergence. High CLR and highly divergent genes are marked by name.

Supplementary Figure S22

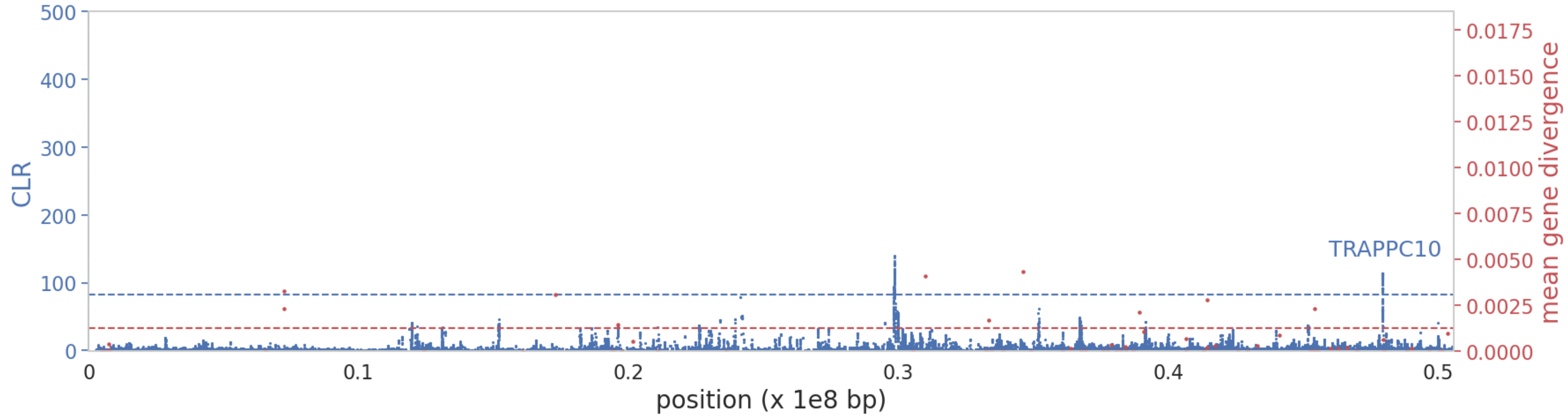

Supplementary Figure S22: SweepFinder2 selective sweep scan results (blue) and exonic divergence (red) for chromosome **21**. The x-axis represents the position along the chromosome, and the y-axis represents the CLR value at each SNP. Blue dashed line represents the null threshold for sweep detection; red dashed line represents 75th percentile neutral divergence. High CLR and highly divergent genes are marked by name.

Supplementary Figure S23

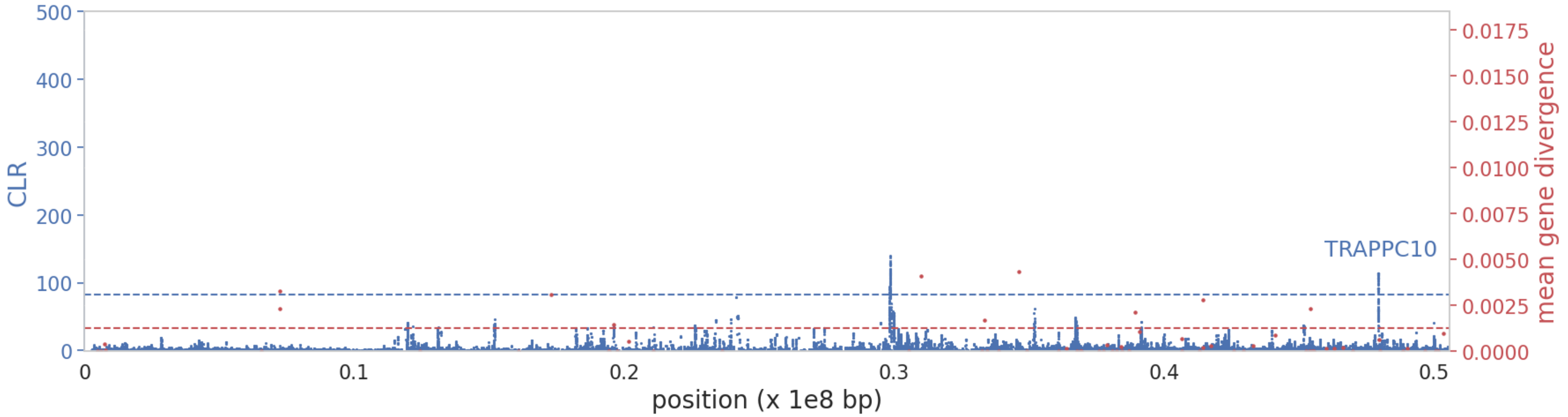

Supplementary Figure S23: SweepFinder2 selective sweep scan results (blue) and exonic divergence (red) for chromosome **22**. The x-axis represents the position along the chromosome, and the y-axis represents the CLR value at each SNP. Blue dashed line represents the null threshold for sweep detection; red dashed line represents 75th percentile neutral divergence. High CLR and highly divergent genes are marked by name.

Supplementary Figure S24

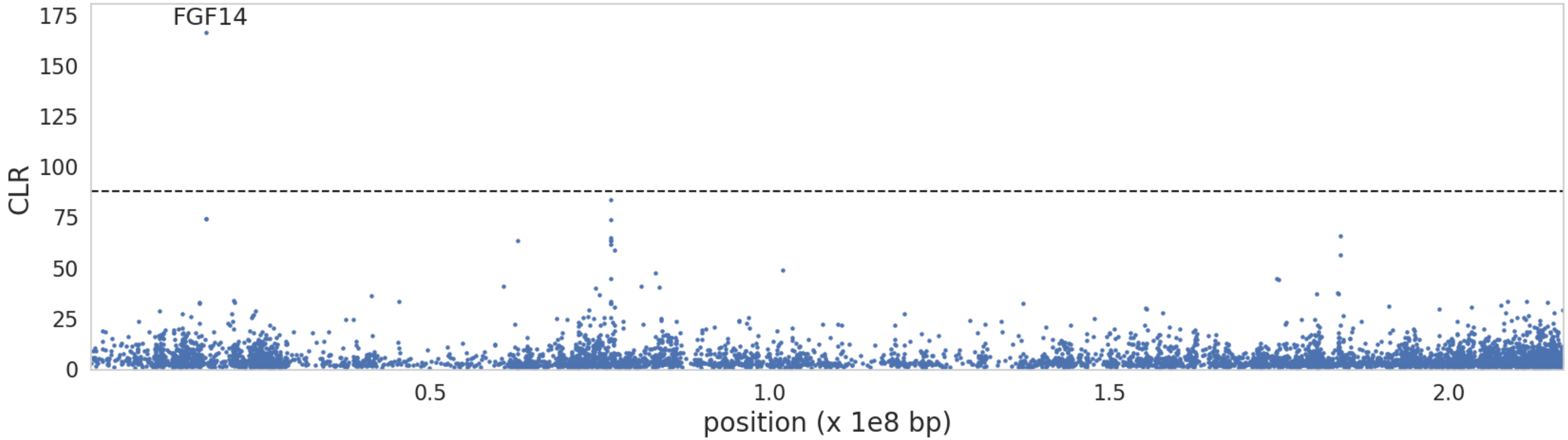

Supplementary Figure S24: B_{0MAF} balancing selection scan results for chromosome **1**. The x-axis represents the position along the chromosome, and the y-axis represents the CLR value at each 1kb window (with a 50bp step size). Black dashed line represents the null threshold for balancing selection inference. Candidate genes are labelled on plot.

Supplementary Figure S25

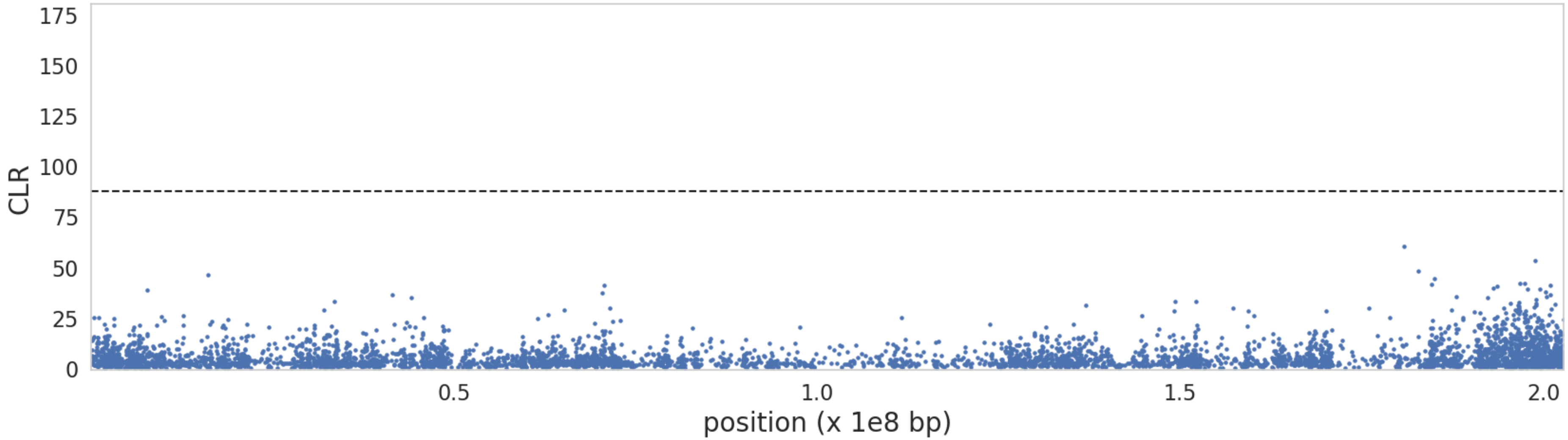

Supplementary Figure S25: B_{0MAF} balancing selection scan results for chromosome **2**. The x-axis represents the position along the chromosome, and the y-axis represents the CLR value at each 1kb window (with a 50bp step size). Black dashed line represents the null threshold for balancing selection inference. Candidate genes are labelled on plot.

Supplementary Figure S26

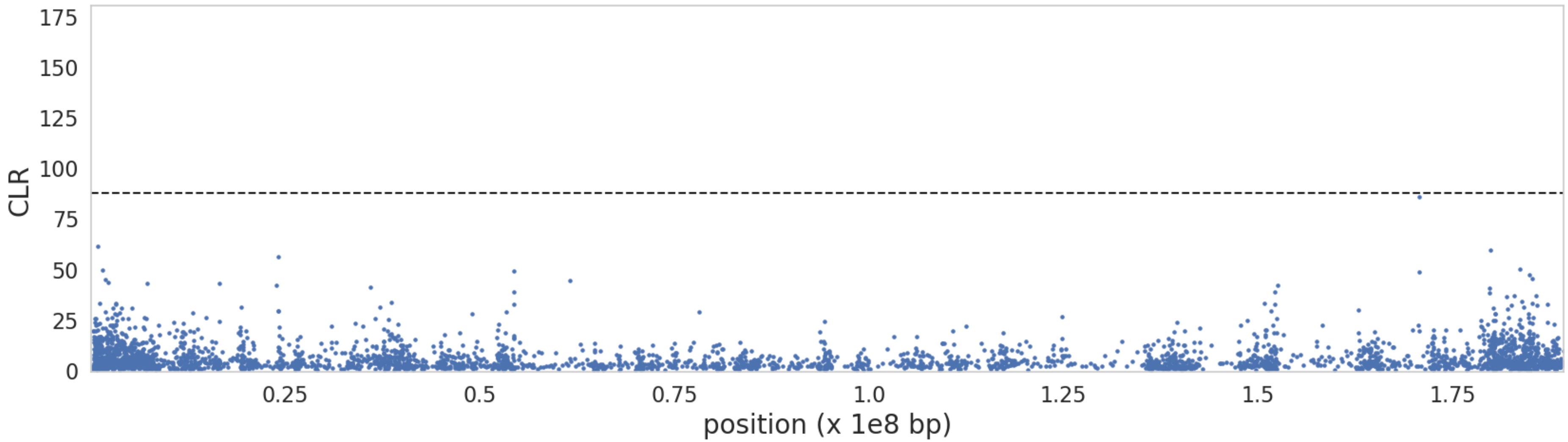

Supplementary Figure S26: B_{0MAF} balancing selection scan results for chromosome **3**. The x-axis represents the position along the chromosome, and the y-axis represents the CLR value at each 1kb window (with a 50bp step size). Black dashed line represents the null threshold for balancing selection inference. Candidate genes are labelled on plot.

Supplementary Figure S27

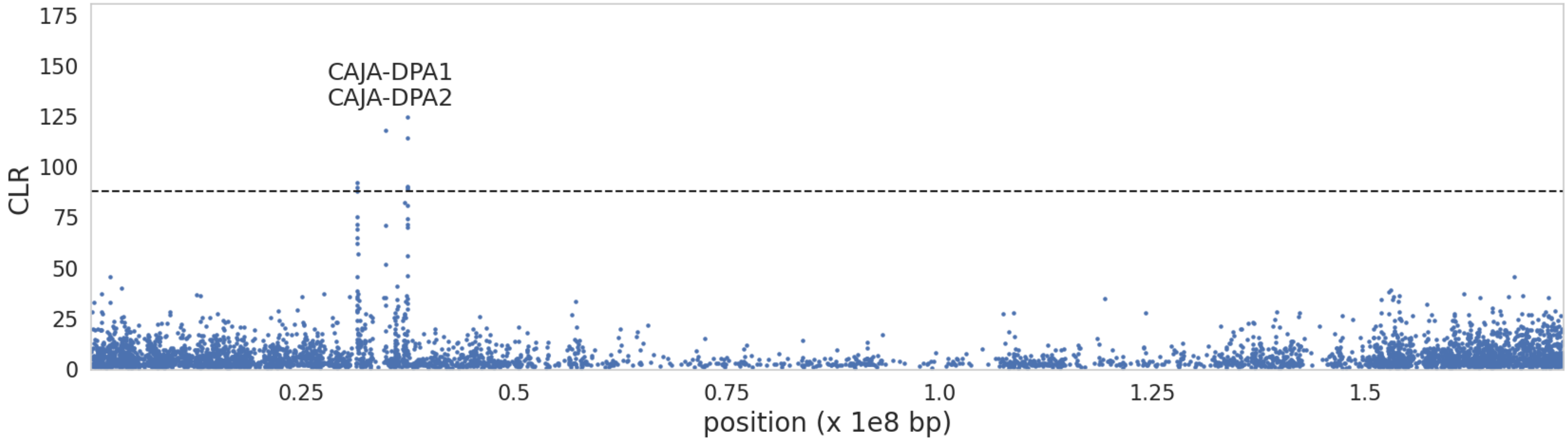

Supplementary Figure S27: B_{0MAF} balancing selection scan results for chromosome **4**. The x-axis represents the position along the chromosome, and the y-axis represents the CLR value at each 1kb window (with a 50bp step size). Black dashed line represents the null threshold for balancing selection inference. Candidate genes are labelled on plot.

Supplementary Figure S28

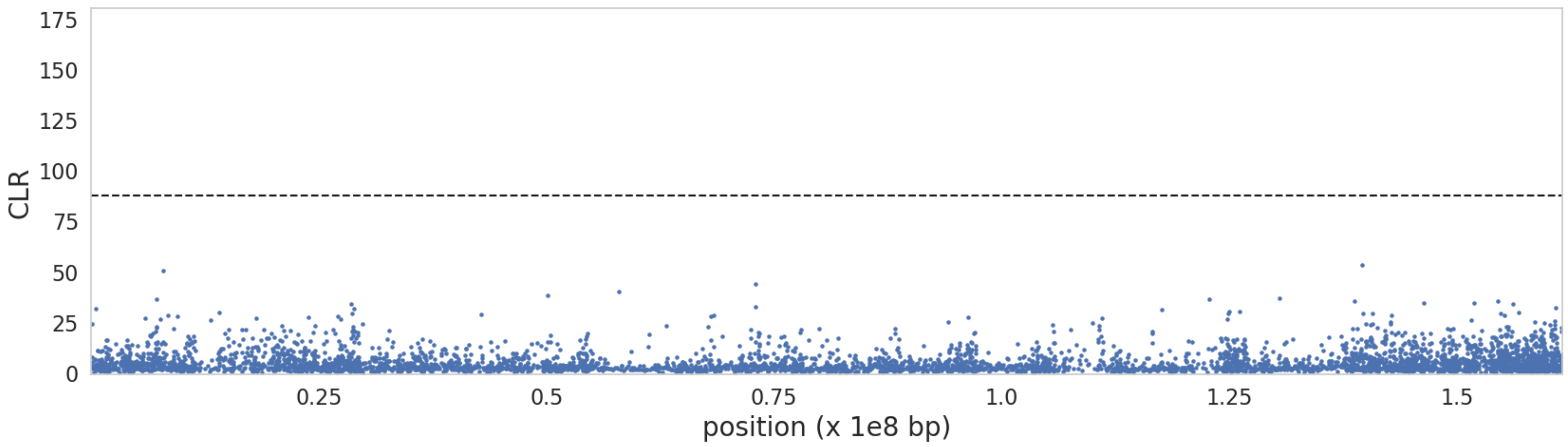

Supplementary Figure S28: B_{0MAF} balancing selection scan results for chromosome **5**. The x-axis represents the position along the chromosome, and the y-axis represents the CLR value at each 1kb window (with a 50bp step size). Black dashed line represents the null threshold for balancing selection inference. Candidate genes are labelled on plot.

Supplementary Figure S29

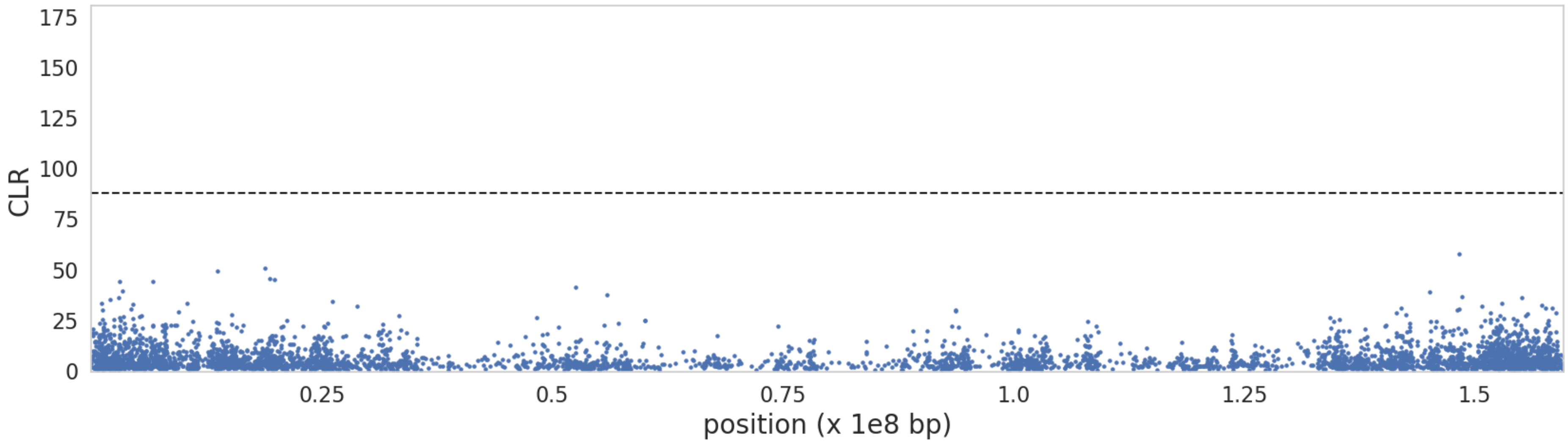

Supplementary Figure S29: B_{0MAF} balancing selection scan results for chromosome **6**. The x-axis represents the position along the chromosome, and the y-axis represents the CLR value at each 1kb window (with a 50bp step size). Black dashed line represents the null threshold for balancing selection inference. Candidate genes are labelled on plot.

Supplementary Figure S30

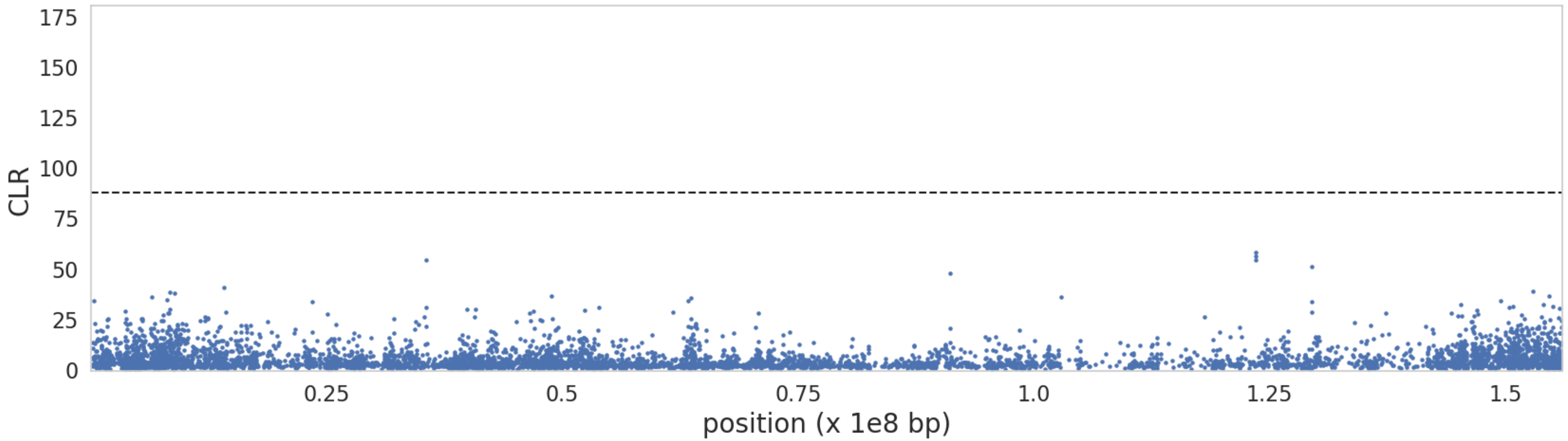

Supplementary Figure S30: B_{0MAF} balancing selection scan results for chromosome **7**. The x-axis represents the position along the chromosome, and the y-axis represents the CLR value at each 1kb window (with a 50bp step size). Black dashed line represents the null threshold for balancing selection inference. Candidate genes are labelled on plot.

Supplementary Figure S31

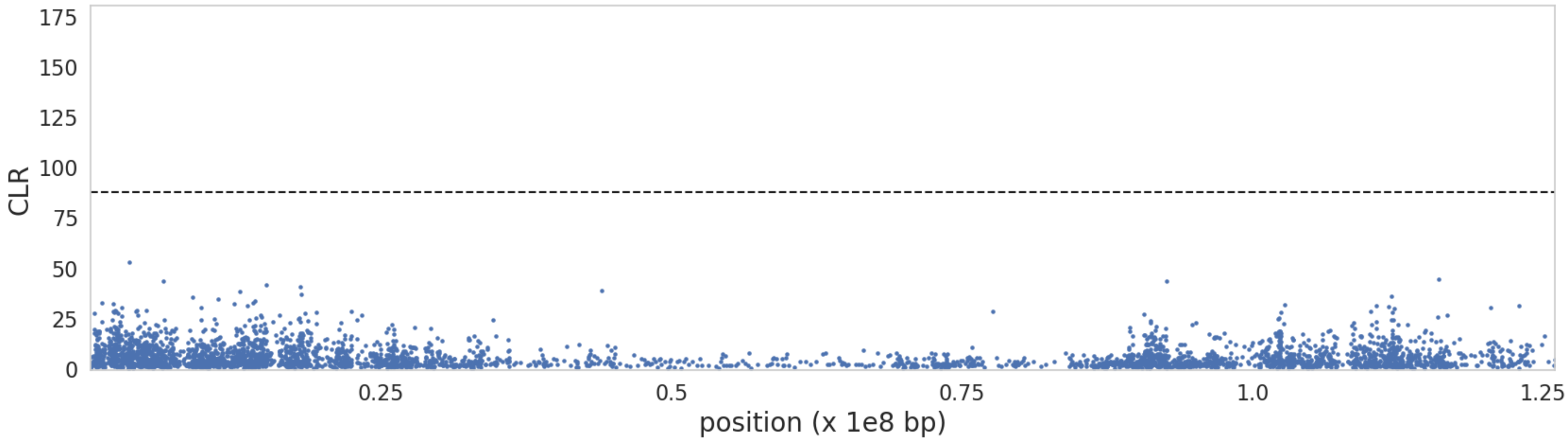

Supplementary Figure S31: B_{0MAF} balancing selection scan results for chromosome **8**. The x-axis represents the position along the chromosome, and the y-axis represents the CLR value at each 1kb window (with a 50bp step size). Black dashed line represents the null threshold for balancing selection inference. Candidate genes are labelled on plot.

Supplementary Figure S32

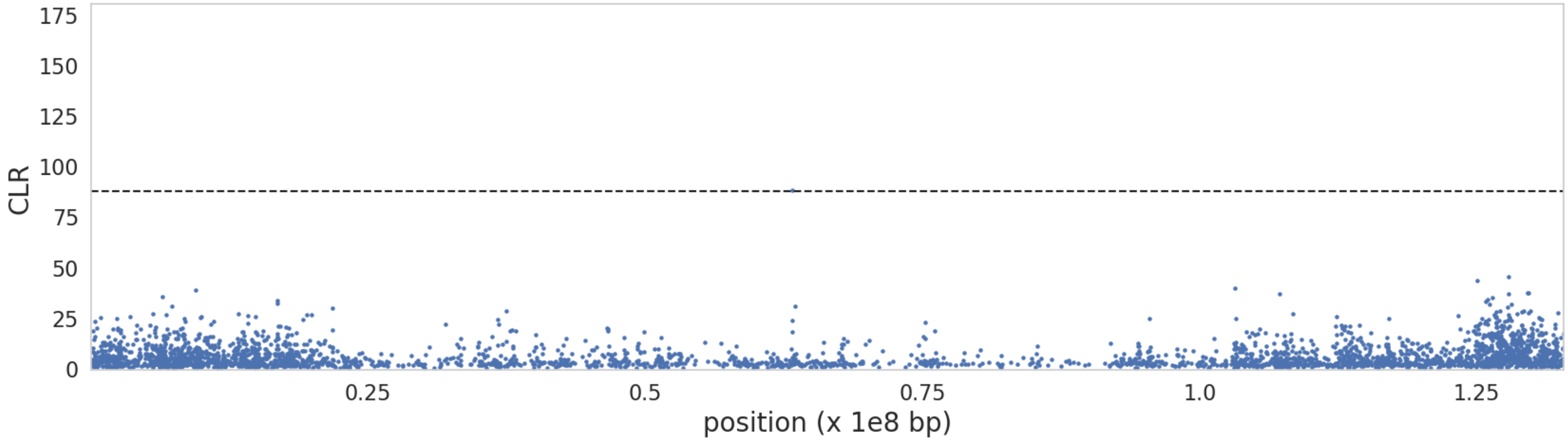

Supplementary Figure S32: B_{0MAF} balancing selection scan results for chromosome **9**. The x-axis represents the position along the chromosome, and the y-axis represents the CLR value at each 1kb window (with a 50bp step size). Black dashed line represents the null threshold for balancing selection inference. Candidate genes are labelled on plot.

Supplementary Figure S33

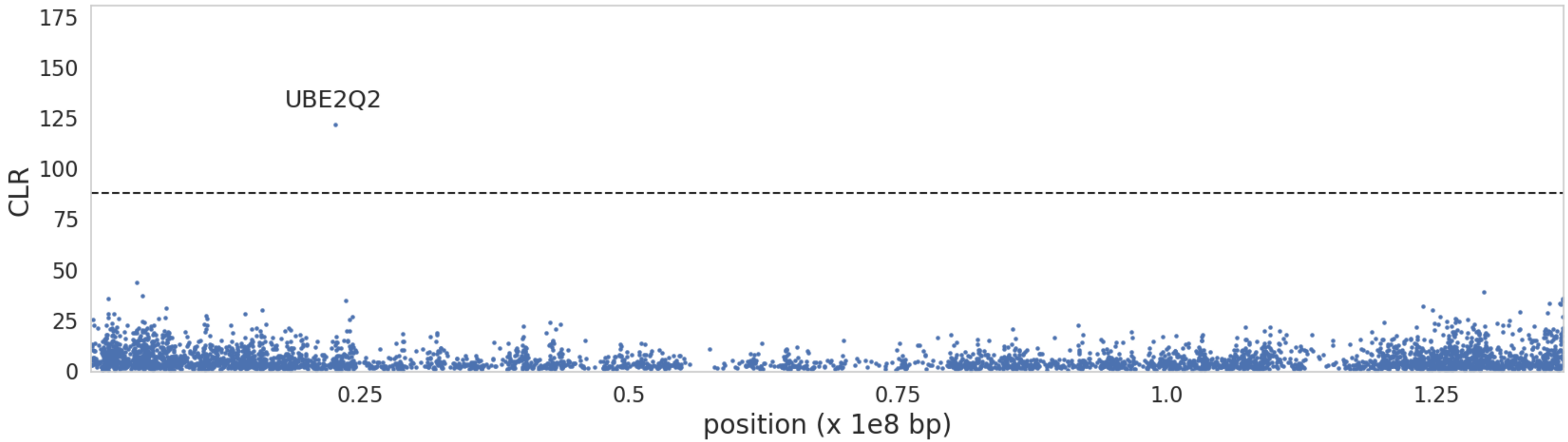

Supplementary Figure S33: B_{0MAF} balancing selection scan results for chromosome **10**. The x-axis represents the position along the chromosome, and the y-axis represents the CLR value at each 1kb window (with a 50bp step size). Black dashed line represents the null threshold for balancing selection inference. Candidate genes are labelled on plot.

Supplementary Figure S34

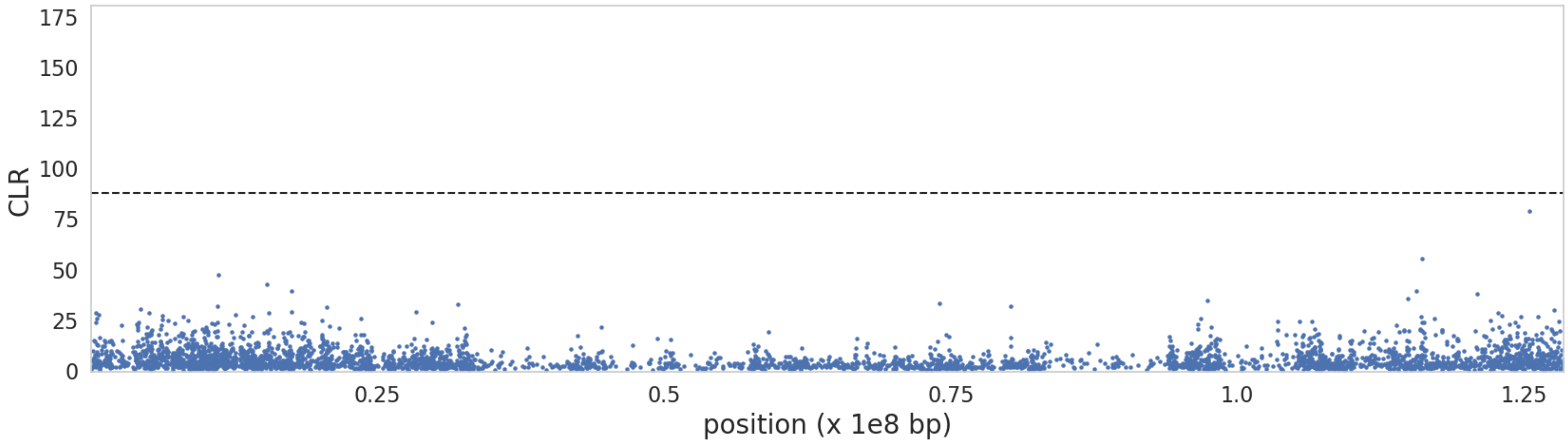

Supplementary Figure S34: B_{0MAF} balancing selection scan results for chromosome **11**. The x-axis represents the position along the chromosome, and the y-axis represents the CLR value at each 1kb window (with a 50bp step size). Black dashed line represents the null threshold for balancing selection inference. Candidate genes are labelled on plot.

Supplementary Figure S35

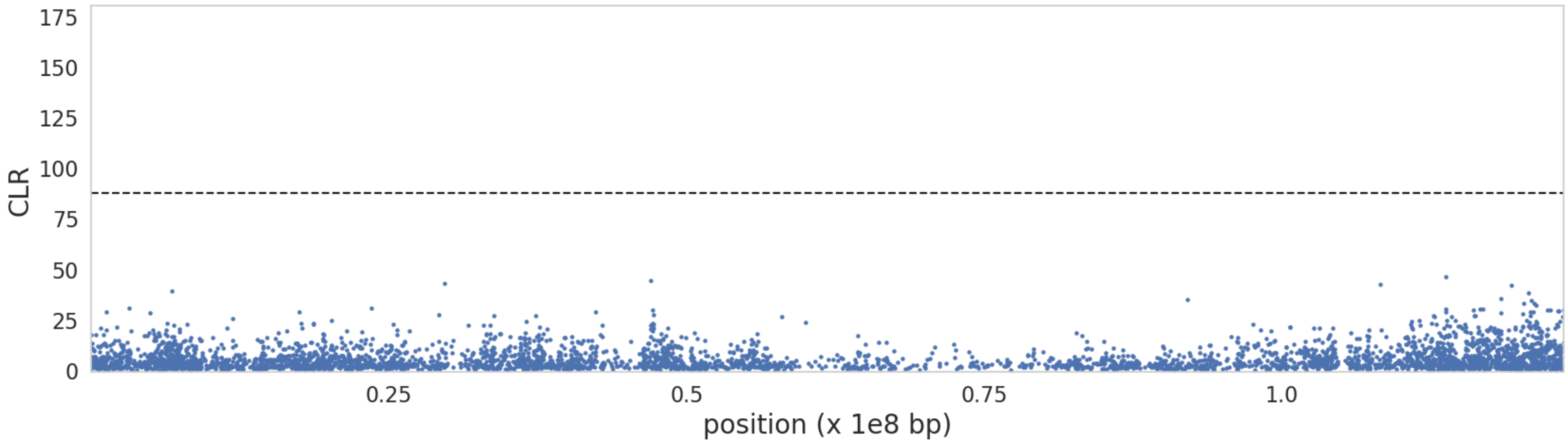

Supplementary Figure S35: B_{0MAF} balancing selection scan results for chromosome **12**. The x-axis represents the position along the chromosome, and the y-axis represents the CLR value at each 1kb window (with a 50bp step size). Black dashed line represents the null threshold for balancing selection inference. Candidate genes are labelled on plot.

Supplementary Figure S36

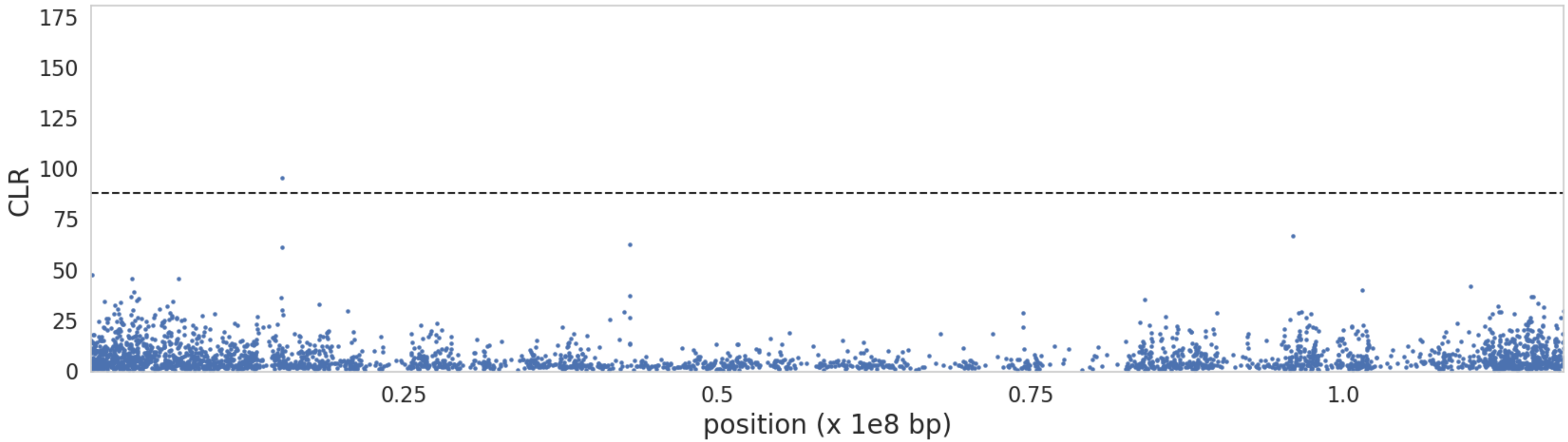

Supplementary Figure S36: B_{0MAF} balancing selection scan results for chromosome **13**. The x-axis represents the position along the chromosome, and the y-axis represents the CLR value at each 1kb window (with a 50bp step size). Black dashed line represents the null threshold for balancing selection inference. Candidate genes are labelled on plot.

Supplementary Figure S37

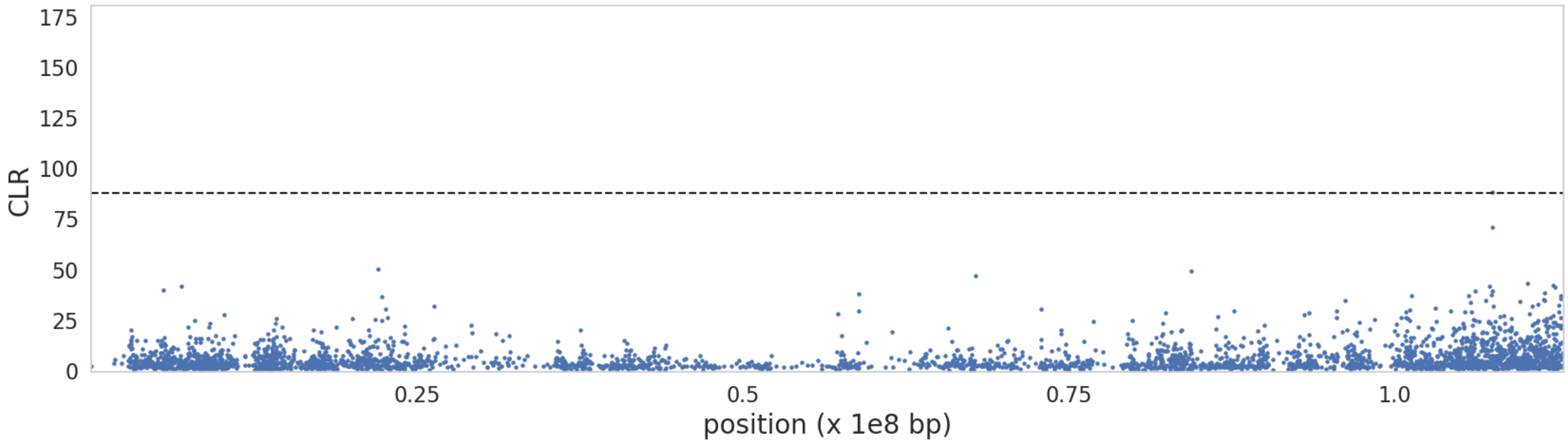

Supplementary Figure S37: B_{0MAF} balancing selection scan results for chromosome **14**. The x-axis represents the position along the chromosome, and the y-axis represents the CLR value at each 1kb window (with a 50bp step size). Black dashed line represents the null threshold for balancing selection inference. Candidate genes are labelled on plot.

Supplementary Figure S38

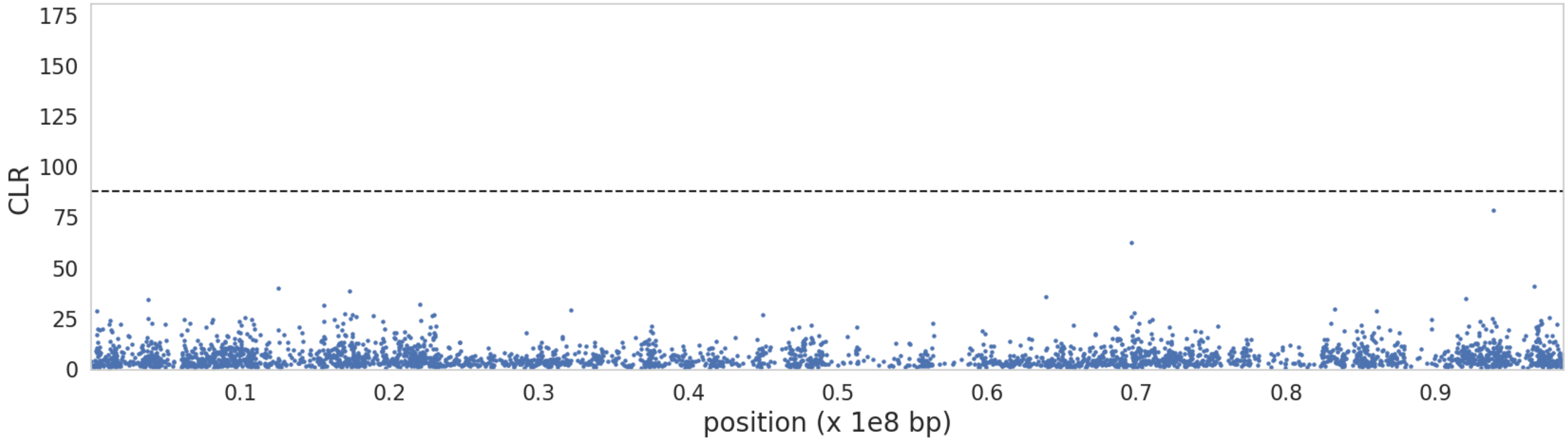

Supplementary Figure S38: B_{0MAF} balancing selection scan results for chromosome **15**. The x-axis represents the position along the chromosome, and the y-axis represents the CLR value at each 1kb window (with a 50bp step size). Black dashed line represents the null threshold for balancing selection inference. Candidate genes are labelled on plot.

Supplementary Figure S39

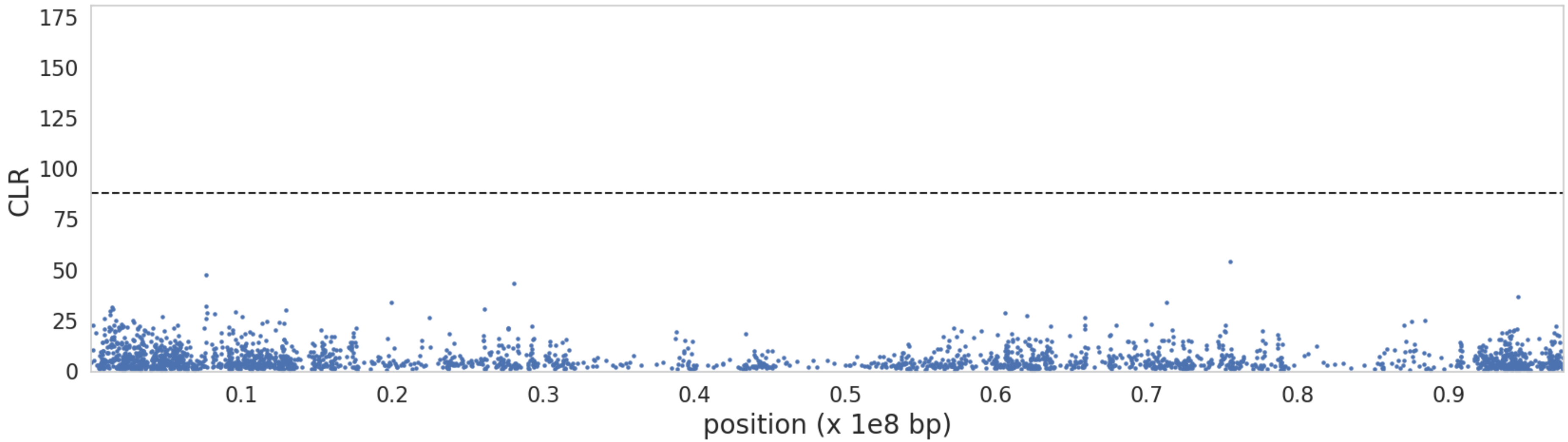

Supplementary Figure S39: B_{0MAF} balancing selection scan results for chromosome **16**. The x-axis represents the position along the chromosome, and the y-axis represents the CLR value at each 1kb window (with a 50bp step size). Black dashed line represents the null threshold for balancing selection inference. Candidate genes are labelled on plot.

Supplementary Figure S40

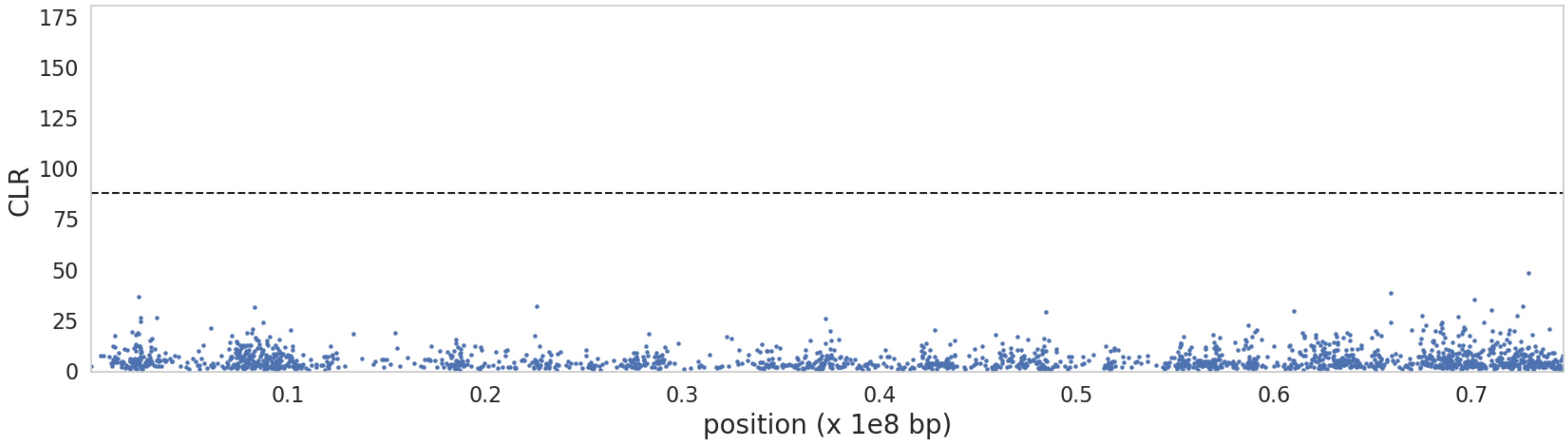

Supplementary Figure S40: B_{0MAF} balancing selection scan results for chromosome **17**. The x-axis represents the position along the chromosome, and the y-axis represents the CLR value at each 1kb window (with a 50bp step size). Black dashed line represents the null threshold for balancing selection inference. Candidate genes are labelled on plot.

Supplementary Figure S41

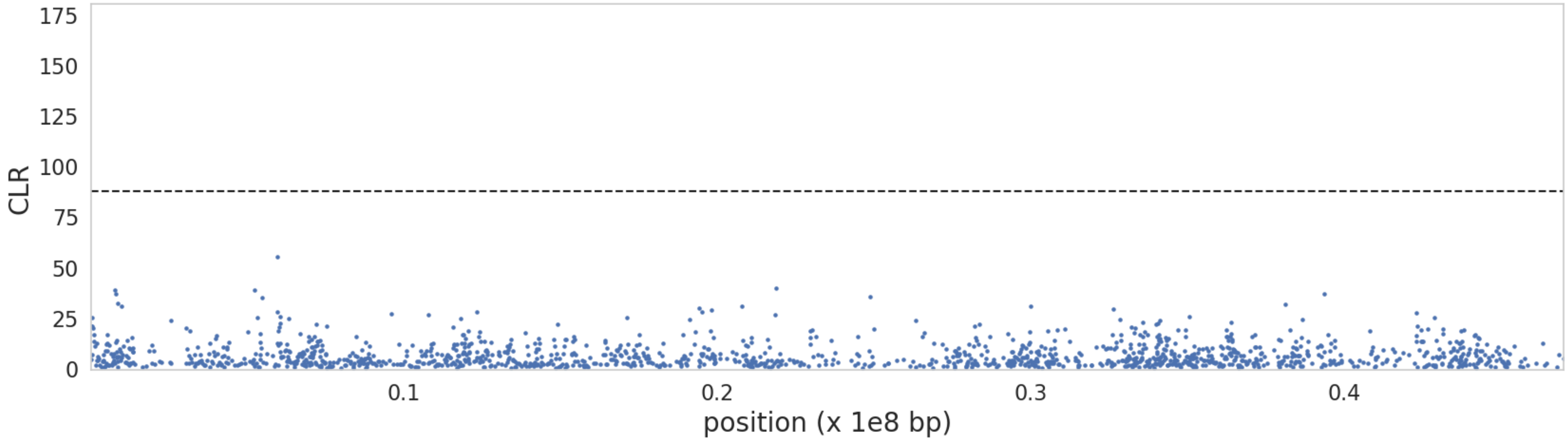

Supplementary Figure S41: B_{0MAF} balancing selection scan results for chromosome **18**. The x-axis represents the position along the chromosome, and the y-axis represents the CLR value at each 1kb window (with a 50bp step size). Black dashed line represents the null threshold for balancing selection inference. Candidate genes are labelled on plot.

Supplementary Figure S42

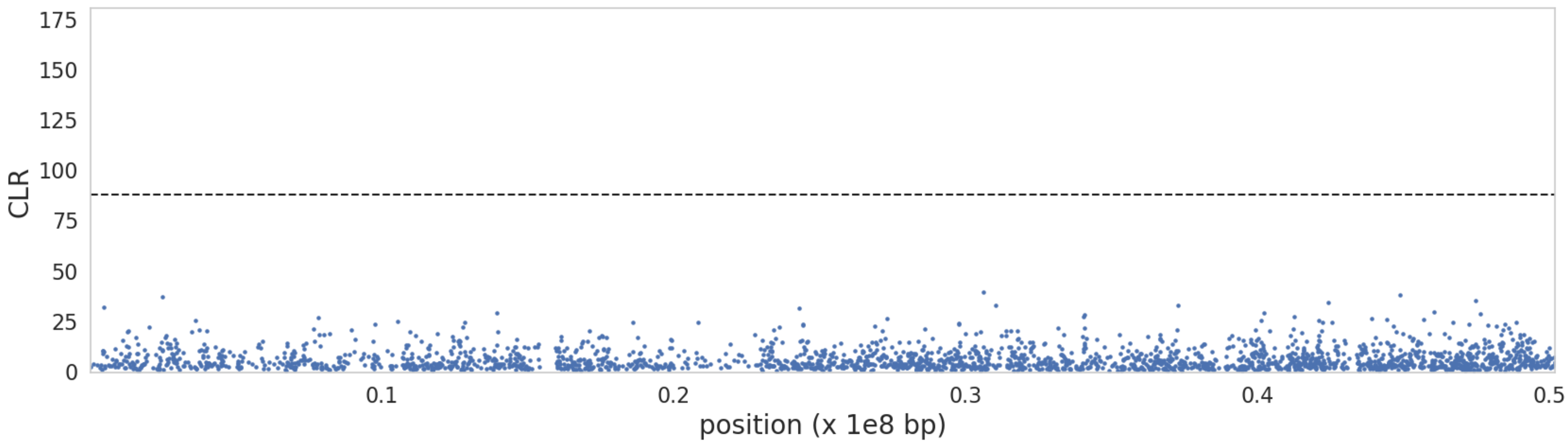

Supplementary Figure S42: B_{0MAF} balancing selection scan results for chromosome **19**. The x-axis represents the position along the chromosome, and the y-axis represents the CLR value at each 1kb window (with a 50bp step size). Black dashed line represents the null threshold for balancing selection inference. Candidate genes are labelled on plot.

Supplementary Figure S43

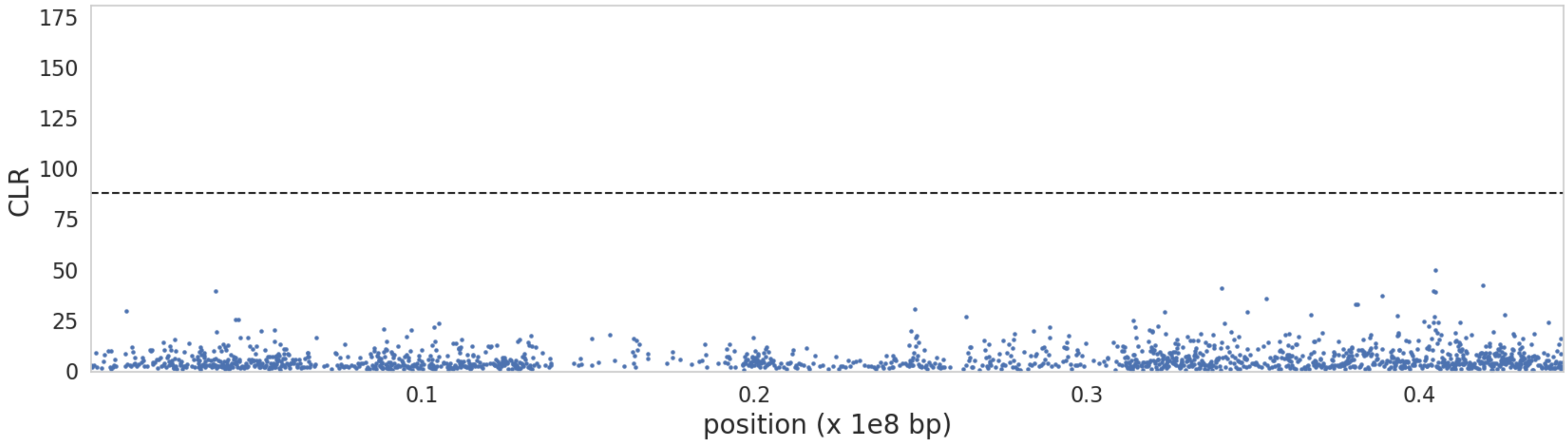

Supplementary Figure S43: B_{0MAF} balancing selection scan results for chromosome **20**. The x-axis represents the position along the chromosome, and the y-axis represents the CLR value at each 1kb window (with a 50bp step size). Black dashed line represents the null threshold for balancing selection inference. Candidate genes are labelled on plot.

Supplementary Figure S44

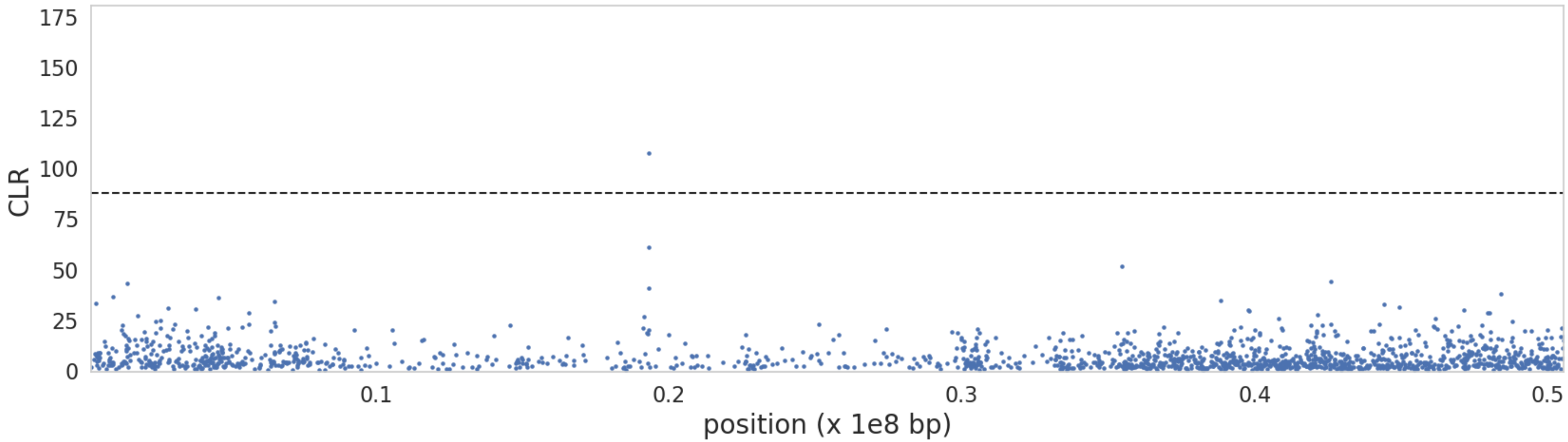

Supplementary Figure S44: B_{0MAF} balancing selection scan results for chromosome **21**. The x-axis represents the position along the chromosome, and the y-axis represents the CLR value at each 1kb window (with a 50bp step size). Black dashed line represents the null threshold for balancing selection inference. Candidate genes are labelled on plot.

Supplementary Figure S45

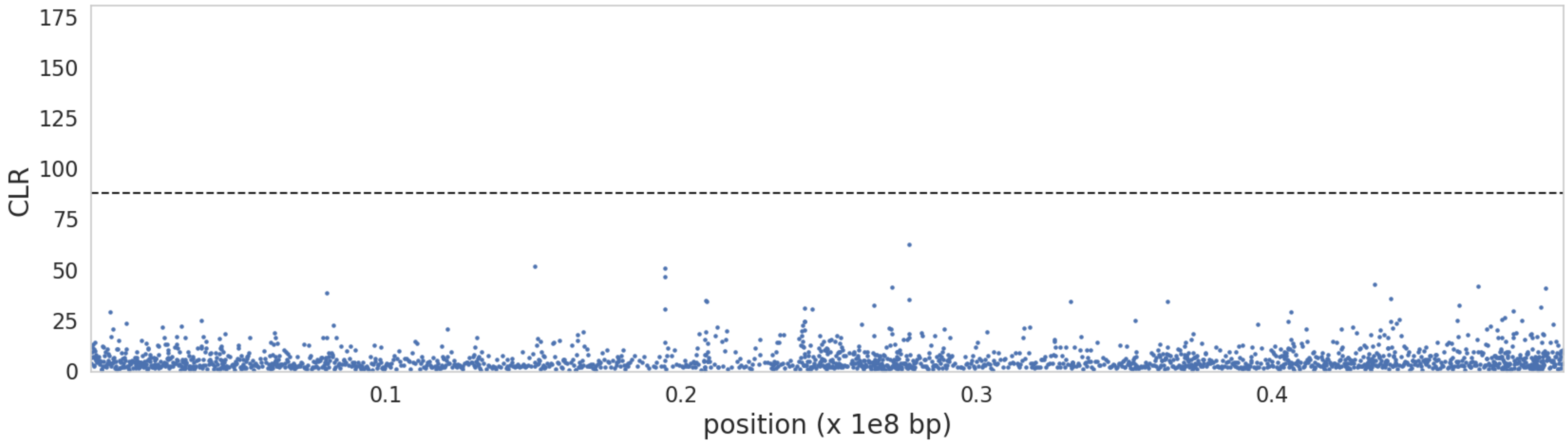

Supplementary figure S45: B_{0MAF} balancing selection scan results for chromosome **22**. The x-axis represents the position along the chromosome, and the y-axis represents the CLR value at each 1kb window (with a 50bp step size). Black dashed line represents the null threshold for balancing selection inference. Candidate genes are labelled on plot.
